# Single‐Mitochondrion ATP Profiling Directs Discovery of Targetable OXPHOS Dependency in Cancers

**DOI:** 10.1002/advs.202513341

**Published:** 2026-02-13

**Authors:** Xu Xiao, Cheng Lu, Hao Chen, Jing Zhou, Yunyun Hu, Haonan Di, Guoqiang Su, Xiaomei Yan

**Affiliations:** ^1^ Department of Chemical Biology MOE Key Laboratory of Spectrochemical Analysis & Instrumentation Fujian Key Laboratory of Chemical Biology (Xiamen University) State Key Laboratory of Physical Chemistry of Solid Surfaces Collaborative Innovation Center of Chemistry for Energy Materials College of Chemistry and Chemical Engineering Xiamen University Xiamen Fujian China; ^2^ Department of Colorectal Tumor Surgery Xiamen Key Laboratory of Early Cancer Diagnosis and Treatment School of Medicine The First Affiliated Hospital of Xiamen University Xiamen University Xiamen Fujian China

**Keywords:** cancer vulnerability, mitochondrial ATP, mitochondrial metabolism, precision cancer therapy, single‐organelle analysis

## Abstract

Mitochondrial adenosine triphosphate (mitoATP) serves as the primary bioenergetic currency for oxidative phosphorylation (OXPHOS)‐driven malignancies, yet its precise organelle‐level quantification remains challenging due to mitochondrial heterogeneity and cytosolic interference. Herein, we report MitoATP‐nFCM, a nano‐flow cytometry platform enabling single‐mitochondrion ATP measurement via simultaneous fluorescence and side scatter detection. We uncover 1.7–1.9‐fold higher ATP levels in isolated mitochondria from breast (MCF‐7, MDA‐MB‐231) and colon (HCT‐15, HCT‐116) cancer cells than in their normal counterparts. Single‐organelle analysis further reveals coordinated metabolic reprogramming in cancer mitochondria, featuring elevated membrane potential, increased ATP synthase expression, and reduced hexokinase 2 levels, demonstrating their OXPHOS‐dominant bioenergetic phenotype that contrasts with classical Warburg‐effect expectations. Furthermore, we establish a screening strategy to identify highly potent cancer‐selective inhibitors targeting mitochondrial metabolism. We find that bedaquiline (ATP synthase inhibitor) outperforms oligomycin A in specificity, VLX600 (electron transport chain inhibitor) shows superior selectivity to rotenone/metformin, and CPI‐613 (tricarboxylic acid cycle blocker) surpasses other glutaminase inhibitors. MitoATP‐nFCM establishes a quantitative single‐organelle platform that profiles elevated mitoATP levels in cancer cells and enables precision screening of OXPHOS‐targeting inhibitors.

## Graphical abstract

MitoATP‐nFCM integrates nano‐flow cytometry with fluorogenic probes (ATP/membrane potential) and antibodies to quantify mitochondrial metabolites and protein expression at single‐organelle resolution, exposing oxidative phosphorylation (OXPHOS)‐driven metabolic rewiring in cancers. Beyond mapping cancer‐specific mitochondrial vulnerabilities, the platform enables precision targeting of inhibitors (bedaquiline/VLX600/CPI‐613) via ATP‐based screening, bridging organelle‐resolved phenotyping to therapeutic discovery.

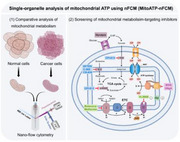

## 1 Introduction

Accumulating clinical and biochemical evidence has established mitochondrial metabolic reprogramming as a hallmark of cancer progression, where dysregulated energy metabolism frequently accompanies malignant transformation [1, 2, 3, 4, 5, 6, 7]. Critically, specific cancer subtypes evolve increased dependence on mitochondrial oxidative phosphorylation (OXPHOS) to sustain elevated mitochondrial adenosine triphosphate (ATP) levels, a metabolic adaptation directly fueling tumor proliferation, metastasis, and chemoresistance [8, 9, 10]. While the Warburg effect highlights a glycolytic phenotype, emerging evidence demonstrates that metabolic stressors like acidosis can induce a shift to mitochondrial respiration [11]. This plasticity is epitomized during metastasis, where in vivo isotope tracing confirms the active upregulation of oxidative phosphorylation, thereby contesting the universality of glycolysis in advanced cancers [12]. Thus, targeting mitochondrial metabolism to deplete mitochondrial ATP (mitoATP) levels emerges as a promising anticancer strategy [13, 14, 15, 16]. However, its implementation faces fundamental measurement barriers. The traditional assays based on ATP‐dependent luciferin‐luciferase reaction introduce artifacts due to destructive sample processing [17]. The utility of fluorescent probes is compromised by both intercompartmental signal crosstalk and the inherent resolution limit (>200 nm axial resolution) of conventional fluorescence microscopy, rendering single‐mitochondrion analysis unachievable [18]. On the other hand, the inherent heterogeneity of mitochondrial populations, where dynamic fusion‐fission processes create metabolically distinct subpopulations, further complicates accurate assessment [19, 20, 21]. These limitations critically impede drug development, as current platforms fail to distinguish mitochondrial ATP inhibition from cytosolic ATP interference and compound off‐target effects on other organelles [22, 23]. Therefore, precise analytical tools for single‐organelle mitoATP quantification with strict mitochondrial specificity are urgently needed to advance therapies for OXPHOS‐dependent cancers.

**SCHEME 1:**
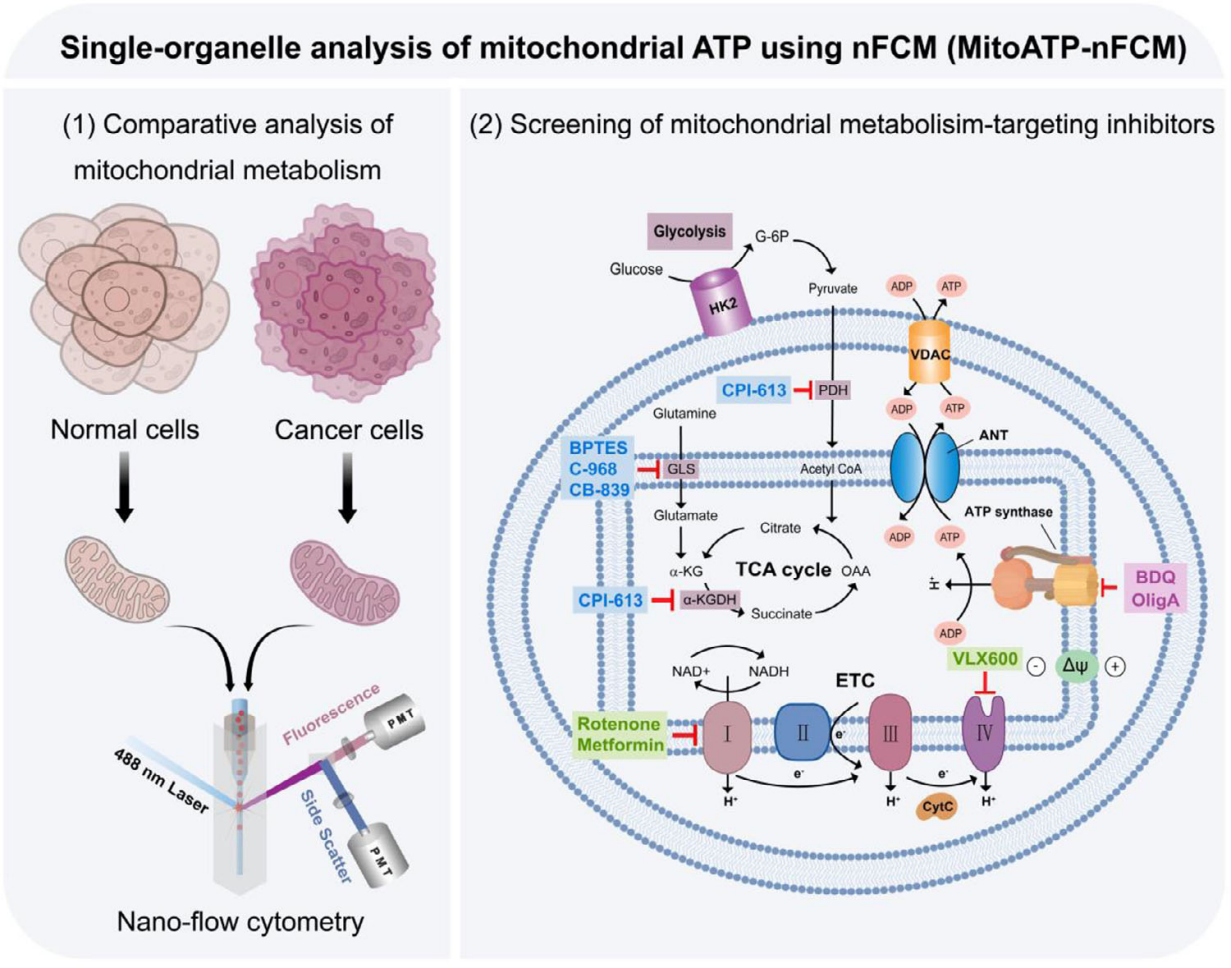
MitoATP‐nFCM: a single‐organelle platform for quantifying cancer‐specific oxidative phosphorylation (OXPHOS) dependency and screening mitochondrial metabolism‐targeted inhibitors.

By integrating a laboratory‐built nano‐flow cytometer (nFCM) [24, 25, 26, 27, 28, 29] with a fluorogenic ATP probe (ATP‐Red 1) [30], we developed MitoATP‐nFCM to resolve a long‐standing paradox in cancer metabolism: while the Warburg effect dominates textbook paradigms, mounting evidence suggests OXPHOS dependency in specific contexts. Our platform enables (1) single‐organelle quantification of mitoATP to definitively map metabolic heterogeneity, revealing coordinated reprogramming features (elevated membrane potential, increased ATP synthase expression, and suppressed hexokinase 2 levels), and (2) precision screening of inhibitors targeting OXPHOS‐dependent cancers, thereby bridging the gap between metabolic phenotyping and targeted therapy (Scheme 1). While the current study focuses on breast and colon cancer models, the MitoATP‐nFCM platform is applicable to other malignancies exhibiting mitochondrial ATP dysregulation.

## 2 Results and Discussion

### 2.1 Development of the MitoATP‐nFCM Platform

**FIGURE 1:**
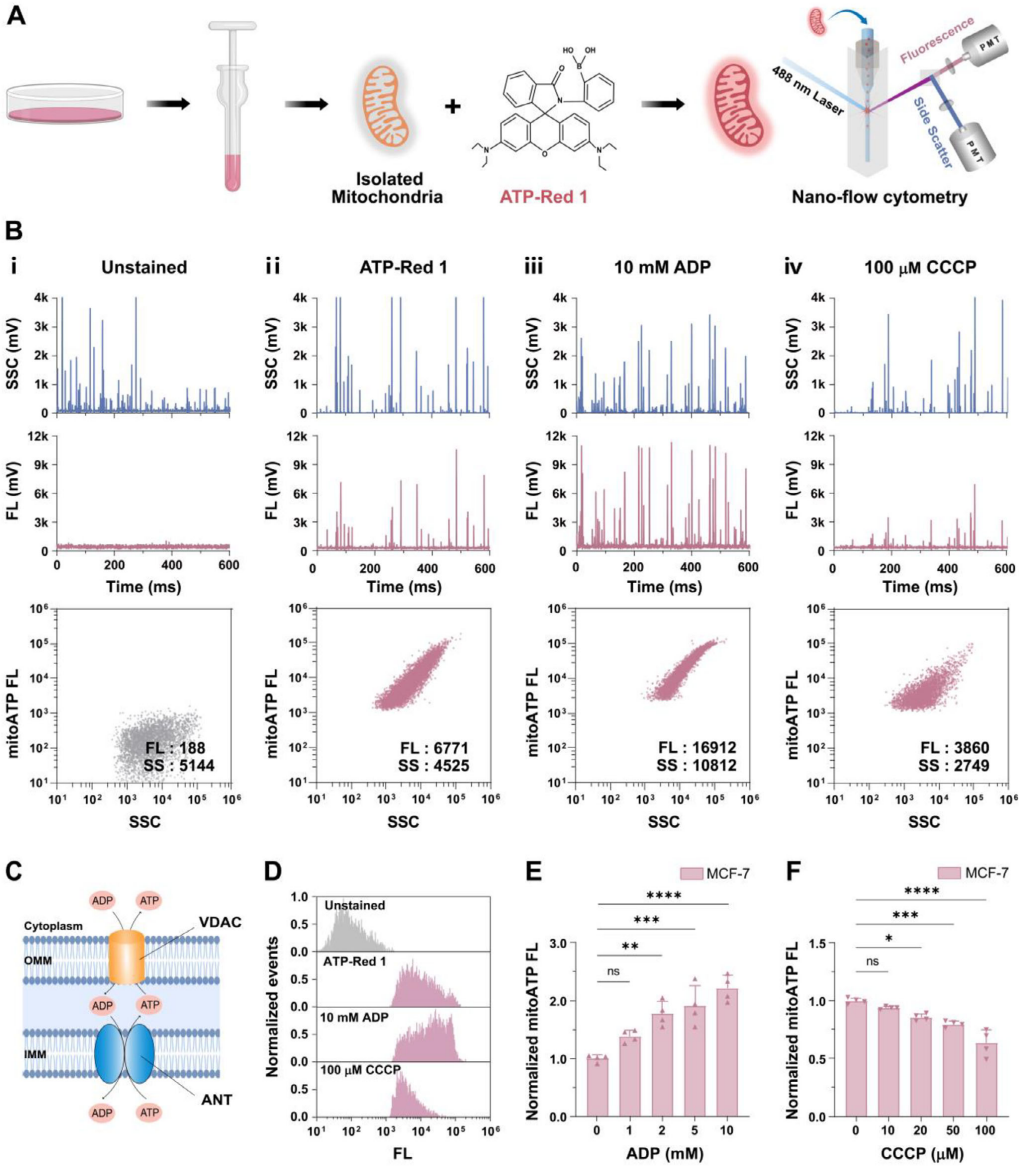
Single‐organelle analysis of mitochondrial ATP using nano‐flow cytometry (MitoATP‐nFCM). (A) Schematic illustration of MitoATP‐nFCM detection principle. (B) Representative side scatter (SSC) and fluorescence (FL) burst traces along with bivariate dot‐plots of mitochondrial ATP (mitoATP) FL burst area versus SSC burst area (60 s acquisition) for mitochondria isolated from MCF‐7 breast cancer cells under four experimental conditions: (i) unstained control, (ii) 20 *µ* m ATP‐Red 1 staining, (iii) post‐10 mm ADP stimulation, and (iv) post‐100 *µ* m CCCP treatment. (C) Schematic representation of mitochondrial ADP/ATP transport mechanism. (D) Histograms showing ATP‐Red 1 FL intensity distribution across the four conditions. (E,F) Quantitative analysis of normalized median mitoATP FL intensity for (E) ADP (0–10 mm, *n* = 4), and (F) CCCP (0–100 *µ* m, *n* = 4). Data represent mean ± standard deviation (SD) from four independent experiments. Statistical significance was determined by one‐way ANOVA with Tukey's test: ns = not significant, ^*^ *p* < 0.05, ^**^ *p* < 0.01, ^***^ *p* < 0.001, ^****^ *p* < 0.0001.

To establish the MitoATP‐nFCM platform, mitochondria isolated from human breast cancer cells (MCF‐7) were incubated with the membrane‐permeable ATP‐Red 1 probe (Figure 1A; Figure S1A,B). The probe showed a linear, dose‐dependent response (R^2^ = 0.9919) to ATP in the physiological concentration range of 1–10 mm [31], and exhibited high selectivity for ATP over adenosine diphosphate (ADP) and adenosine monophosphate (AMP) (Figure S1C,D), as well as good fluorescence stability over time (Figure S1E). Specific detection of ATP at the single‐mitochondrion level was achieved by MitoATP‐nFCM, as demonstrated by a marked fluorescence (FL) increase after ATP‐Red 1 labeling in comparison to controls, accompanied by one‐to‐one correspondence between side scatter (SSC) and FL channels (Figure 1Bi,ii and D). Although live‐cell imaging revealed predominant mitochondrial localization of ATP‐Red 1, minor lysosomal colocalization was observed (Figure S2), demonstrating that our platform circumvented this potential interference using isolated mitochondria. Mitochondrial integrity remained intact during staining, as indicated by stable SSC signals across probe concentrations (0–100 *µ* m) (Figure S3A,B). Saturation of FL intensity at 20 *µ* m ATP‐Red 1 (>90% labeling efficiency) defined the optimal concentration for subsequent experiments (Figure S3C,D).

To validate mitochondrial functionality, isolated mitochondria were treated with ADP or carbonyl cyanide m‐chlorophenyl hydrazone (CCCP), a potent mitochondrial uncoupler, and analyzed using MitoATP‐nFCM. Under respiratory conditions, ADP traverses the outer mitochondrial membrane (OMM) via the voltage‐dependent anion channel (VDAC) and is imported into the matrix by adenine nucleotide translocase (ANT), where it serves as a substrate for ATP synthase (Figure 1C) [32]. MitoATP‐nFCM analysis showed that treatment with 10 mm ADP induced a 2.2‐fold increase in mitoATP FL intensity (Figure 1Bii,iii and D,E), and the dose‐dependent mitoATP FL enhancement across 0–10 mm ADP confirmed preserved OXPHOS functionality in isolated mitochondria (Figure 1E; Figure S4A). Conversely, treatment with 100 *µ* m CCCP reduced mitoATP FL intensity by 43% due to membrane depolarization (Figure 1Bii,iv and D,F), with both dose‐ and time‐dependent effects (Figure 1F; Figures S4B and S5). Finally, to rule out non‐specific binding or interference from structural analogs, isolated mitochondria were incubated with increasing concentrations (0–10 mm) of AMP, a structural analog of ATP that cannot serve as a substrate for ATP synthesis. As shown in Figure S6, AMP treatment did not induce any significant change in the mitoATP fluorescence signal, confirming that the observed probe response is specific to mitochondrial ATP synthesis and not an artifact of non‐specific interaction with organellar components. Altogether, these results indicate that MitoATP‐nFCM enables quantitative, single‐organelle ATP analysis with minimal non‐mitochondrial interference, providing a powerful tool for mitochondria‐targeting drug discovery.

### 2.2 Cancer Cells Exhibit Enhanced OXPHOS Activity at the Single‐Mitochondrion Level

**FIGURE 2:**
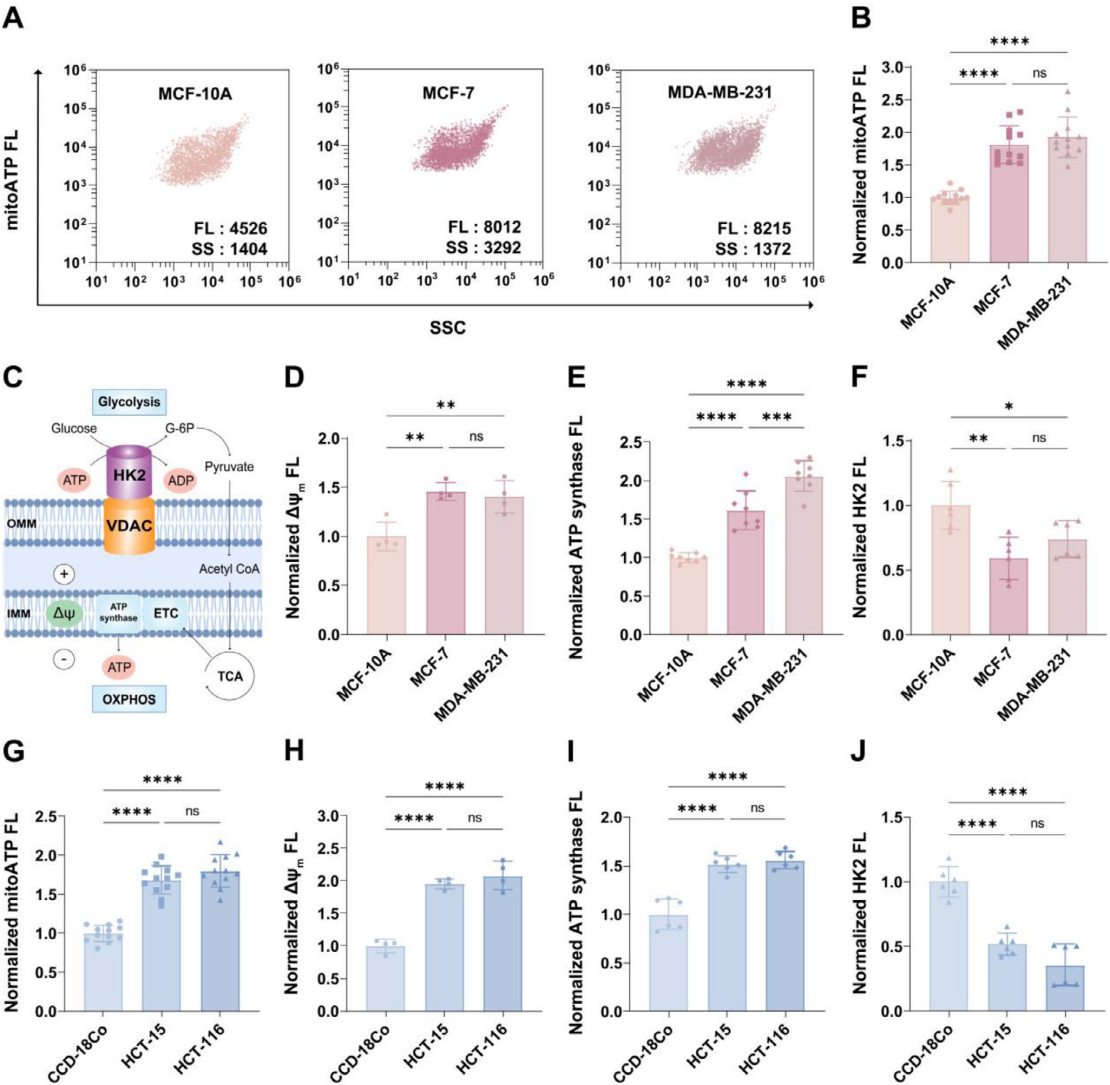
Single‐mitochondrion analysis of metabolic differences between normal cells and cancer cells. (A,B) Mitochondria isolated from breast cell lines (MCF‐10A, MCF‐7, and MDA‐MB‐231) were analyzed using MitoATP‐nFCM. (A) Bivariate dot‐plots of mitoATP FL burst area versus SSC burst area. (B) Normalized median mitoATP FL intensity (*n* = 12). (C) Schematic diagram of OXPHOS versus glycolysis pathways. (D) Normalized median FL intensity of mitochondrial membrane potential (Δ*ψ*_m_) (*n* = 4). (E) Normalized median FL intensity of mitochondrial ATP synthase (*n* = 8). (F) Normalized median FL intensity of glycolytic hexokinase 2 (HK2) (*n* = 6). (G‐J) Corresponding analysis for colorectal cell lines (CCD‐18Co, HCT‐15, and HCT‐116). Normalized median FL intensity of (G) mitoATP (*n* = 12), (H) Δ*ψ*_m_ (*n* = 4), (I) ATP synthase (*n* = 6), (J) HK2 (*n* = 6). Data represent mean ± SD from ≥ 4 independent experiments. Statistical significance was determined by one‐way ANOVA with Tukey's test: ns = not significant, ^*^ *p* < 0.05, ^**^ *p* < 0.01, ^***^ *p* < 0.001, ^****^ *p* < 0.0001.

To investigate the role of mitochondrial metabolism in tumor growth and proliferation, we performed comparative analysis of mitochondria isolated from human mammary immortalized epithelial cells (MCF‐10A), MCF‐7, and triple‐negative breast cancer (TNBC) cells (MDA‐MB‐231) using the MitoATP‐nFCM platform. Strikingly, both cancer cell lines exhibited substantially elevated mitoATP levels, with MCF‐7 showing a 1.8‐fold elevation and MDA‐MB‐231 displaying a 1.9‐fold increase compared to MCF‐10A controls (Figure 2A,B). These quantitative measurements underscore the critical role of mitochondrial OXPHOS in breast cancer progression.

Within mitochondria, the tricarboxylic acid (TCA) cycle, ATP synthase (complex V), and the electron transport chain (ETC) act in concert to drive efficient mitoATP generation [33]. In OXPHOS, mitochondria maintain high membrane potential (Δ*ψ*_m_) via ETC activity to power ATP synthesis [34]. In contrast, glycolysis initiates with hexokinase (HK)‐catalyzed conversion of glucose to glucose‐6‐phosphate (G‐6P) [35]. Among HK isoforms, HK2 binds to the OMM via interaction with VDAC and is in proximity to intramitochondrial ATP, consequently promoting glycolysis [36]. To further validate the observed metabolic shift toward OXPHOS in cancer cells, we conducted single‐organelle measurements of both mitochondrial Δ*ψ*_m_ and protein expression (ATP synthase and HK2) employing nFCM (Figure 2C). Quantitative analysis revealed that cancer mitochondria displayed a 1.4‐fold higher Δ*ψ*_m_ compared to normal controls (Figure 2D; Figure S7A). Furthermore, ATP synthase levels were substantially increased, rising by 61% in MCF‐7 and most notably by 106% in MDA‐MB‐231 relative to MCF‐10A controls (Figure 2E; Figure S7B). Conversely, HK2 protein expression was markedly reduced in malignant cells, declining to 59% of normal levels in MCF‐7 cells and 74% in MDA‐MB‐231 cells (Figure 2F; Figure S7C). These findings conclusively establish that breast cancer cells favor OXPHOS‐dominated bioenergetics over HK2‐mediated glycolytic pathways for their energy requirements.

To investigate whether this organelle‐level metabolic reprogramming could be conserved in other cell lines, we extended MitoATP‐nFCM analysis to mitochondria isolated from normal human colorectal fibroblasts (CCD‐18Co) and two colon cancer cell lines (HCT‐15 and HCT‐116). The results demonstrated remarkable consistency, with MitoATP‐nFCM revealing significantly elevated mitoATP levels in both colon cancer cells compared to normal CCD‐18Co cells, where HCT‐15 and HCT‐116 cells exhibited 1.7‐fold and 1.8‐fold increases respectively (Figure 2G and Figure S8A). This enhanced mitochondrial metabolic activity was further validated by increased Δ*ψ*_m_, upregulated ATP synthase protein expression, and reduced HK2 levels in colon cancer cells (Figure 2H–J; Figure S8B–D).

Of note, conventional flow cytometry detected no significant differences in total cellular ATP content between normal and cancer cells (Figure S9). This observation, combined with our nFCM finding of a ∼1.7‐ to 1.9‐fold increase in ATP levels per individual mitochondrion (Figure 2), suggests a distinct bioenergetic strategy: the enhanced ATP output in cancer cells is likely achieved by boosting the efficiency of existing mitochondria through OXPHOS upregulation, rather than by increasing mitochondrial biogenesis. Taken together, MitoATP‐nFCM reveals that OXPHOS augmentation persists in both breast and colon cancer cells. This conserved metabolic rewiring demonstrates enhanced OXPHOS capacity at the single‐organelle level, suggesting that Warburg‐effect dominance may not apply across all cancer lineages. Importantly, these findings position mitoATP‐depletion as a viable therapeutic strategy for OXPHOS‐dependent cancers.

### 2.3 MitoATP‐nFCM Identifies Cancer‐Specific Vulnerability to ATP Synthase Inhibition

**FIGURE 3:**
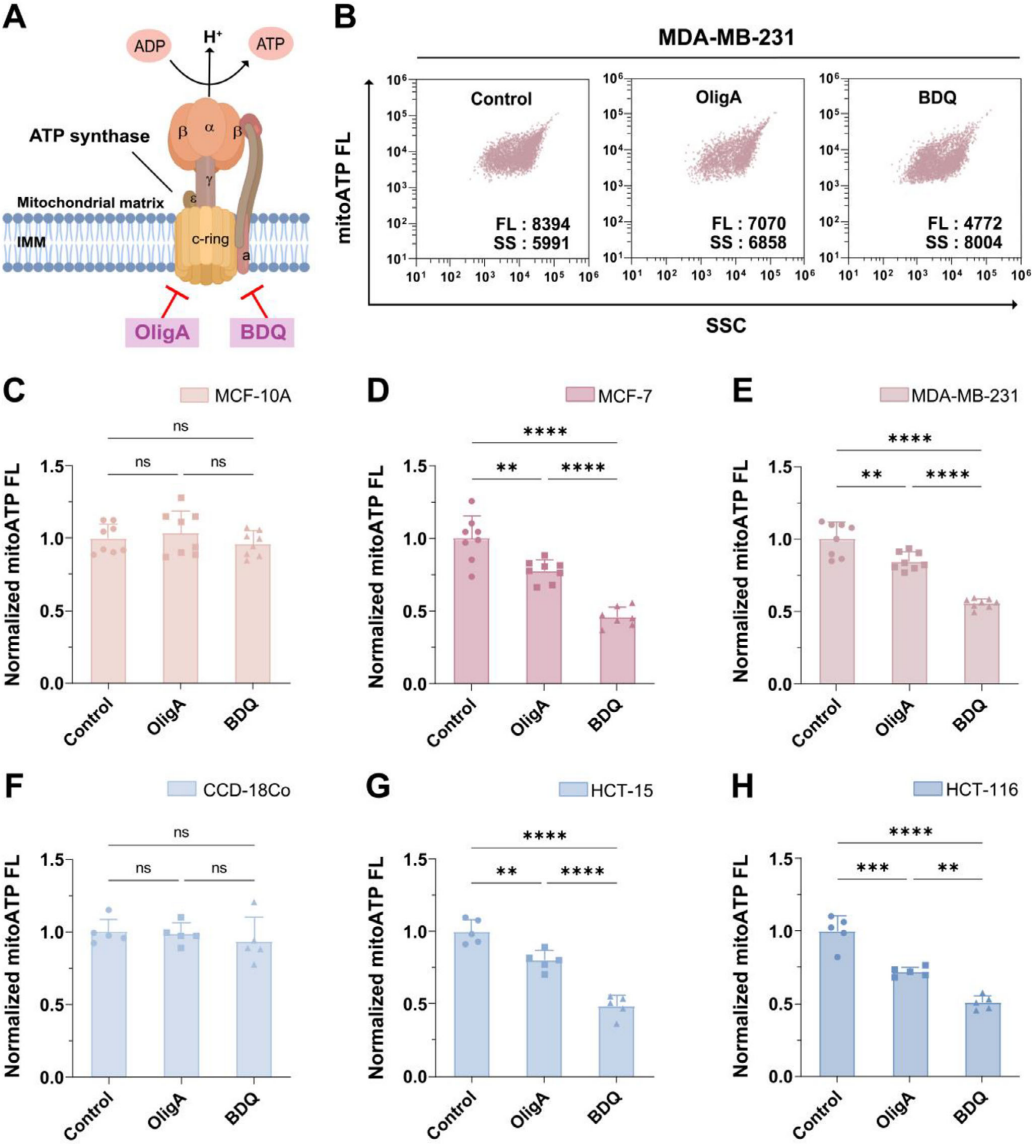
Comparative evaluation of mitochondrial ATP synthase inhibitors using MitoATP‐nFCM. (A) Schematic diagram illustrating the binding sites of oligomycin A (OligA) and bedaquiline (BDQ) on ATP synthase. (B–E) Isolated mitochondria were subjected to MitoATP‐nFCM analysis following treatment with vehicle control, OligA, or BDQ. (B) Bivariate dot‐plots of mitoATP FL burst area versus SSC burst area in MDA‐MB‐231 cells mitochondria. Normalized median mitoATP FL intensity in (C) MCF‐10A (*n* = 8), (D) MCF‐7 (*n* = 8), and (E) MDA‐MB‐231 (*n* = 8) cells mitochondria. (F‐H) Parallel assessment in colon cancer models: normalized median mitoATP FL intensity in (F) CCD‐18Co (*n* = 5), (G) HCT‐15 (*n* = 5), and (H) HCT‐116 (*n* = 5) cells mitochondria. Data represent mean ± SD from ≥ 5 independent experiments. Statistical significance was determined by one‐way ANOVA with Tukey's test: ns = not significant, ^**^ *p* < 0.01, ^***^ *p* < 0.001, ^****^ *p* < 0.0001.

Given our observation of upregulated ATP synthase expression in cancer cells at single‐mitochondrion resolution (Figure 2E,I), we developed a drug screening strategy via MitoATP‐nFCM to explore mitochondrial ATP synthase as a potential anticancer target. We treated MCF‐7 cells with oligomycin A (OligA), a selective inhibitor of the F_0_ subunit c‐ring in ATP synthase, which also induces Δ*ψ*_m_ hyperpolarization, serving as a positive control (Figure 3A). MitoATP‐nFCM analysis revealed a dose‐dependent reduction in mitoATP levels, with 50 *µ* m OligA identified as the optimal concentration for subsequent screening (Figure S10). As expected, OligA treatment induced a dose‐dependent increase in Δ*ψ*_m_ (Figure S11), confirming that MitoATP‐nFCM measurements are independent of Δ*ψ*_m_ alterations.

To compare the therapeutic potential of ATP synthase inhibition in malignant versus normal cells, we assessed OligA alongside bedaquiline (BDQ), an FDA‐approved antimicrobial agent targeting ATP synthase c and a subunits [13, 37]. MitoATP‐nFCM analysis revealed that neither inhibitor altered mitoATP levels in normal MCF‐10A cells, whereas both inhibitors significantly suppressed mitoATP in cancer cells (Figure 3B–E; Figure S12), likely attributable to the higher ATP synthase abundance in malignant cells. Strikingly, BDQ exhibited superior efficacy to OligA, reducing mitoATP to 46% of untreated controls in MCF‐7 and 56% in MDA‐MB‐231 cells (Figure 3D,E), suggesting its cancer‐cell selectivity. This trend also extended to colon cancer models, where BDQ decreased mitoATP by 49% in HCT‐15 and 51% in HCT‐116 relative to untreated controls, respectively (Figure 3F–H; Figure S13). Collectively, our MitoATP‐nFCM platform reveals that cancer cells with higher mitoATP levels exhibit heightened sensitivity to ATP synthase inhibition and underscores the potential of BDQ as a promising mitochondria‐targeted therapeutic agent; importantly, this strong concordance between the observed molecular phenotype and the functional response validates the accuracy and biological relevance of our screening approach.

### 2.4 MitoATP‐nFCM Delineates Cancer‐Selective Electron Transport Chain Inhibition

**FIGURE 4:**
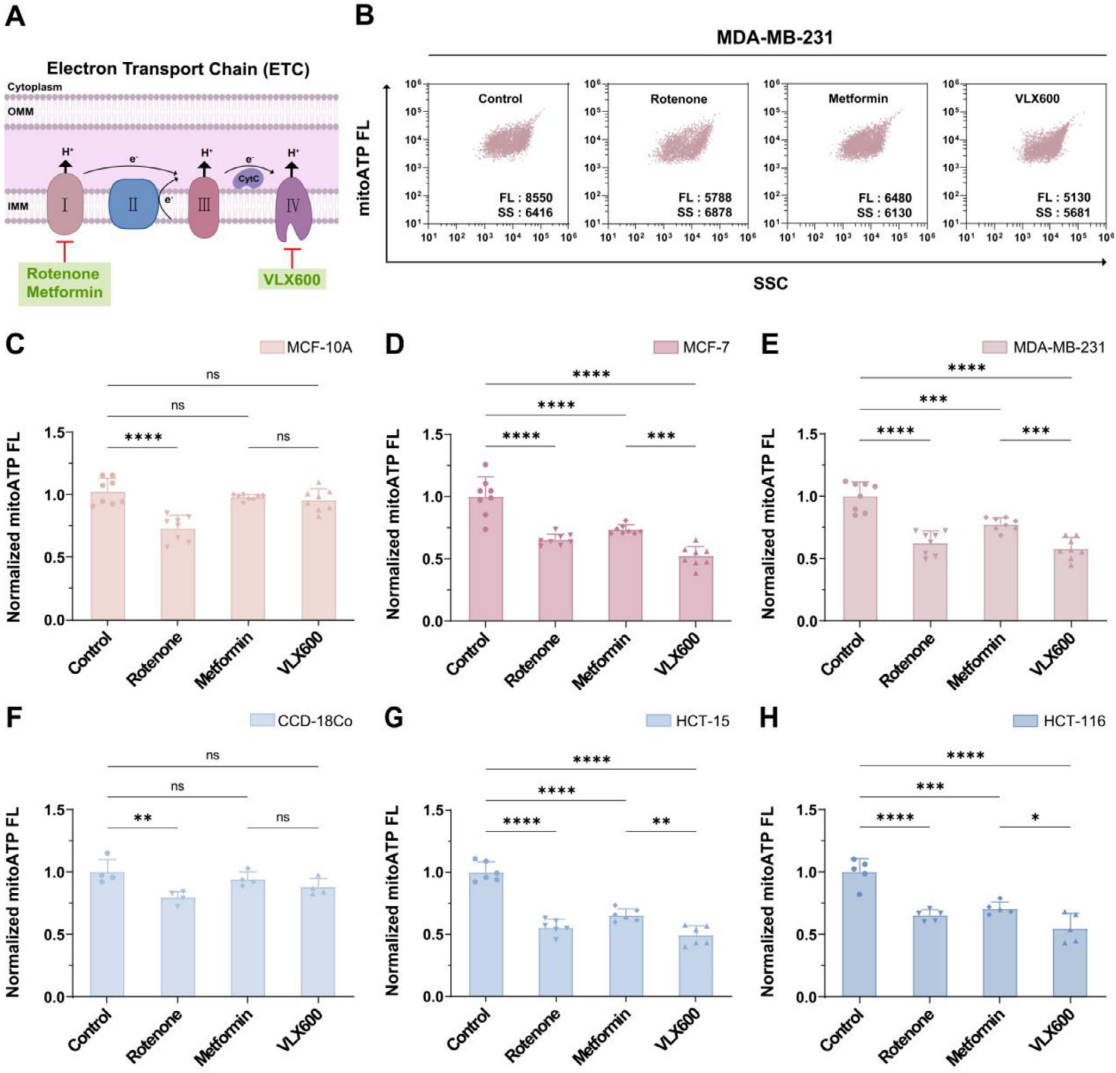
Comparative analysis of electron transport chain (ETC) inhibitors by MitoATP‐nFCM. (A) Schematic diagram illustrating the binding sites of three ETC inhibitors. (B–E) Isolated mitochondria were subjected to MitoATP‐nFCM analysis following treatment with vehicle control, rotenone, metformin, or VLX600. (B) Bivariate dot‐plots of mitoATP FL burst area versus SSC burst area in MDA‐MB‐231 cells mitochondria. Normalized median mitoATP FL intensity in (C) MCF‐10A (*n* = 8), (D) MCF‐7 (*n* = 8), and (E) MDA‐MB‐231 (*n* = 8) cells mitochondria. (F‐H) Parallel assessment in colon cancer models: normalized median mitoATP FL intensity in (F) CCD‐18Co (*n* = 4), (G) HCT‐15 (*n* = 6), and (H) HCT‐116 (*n* = 5) cells mitochondria. Data represent mean ± SD from ≥ 4 independent experiments. Statistical significance was determined by one‐way ANOVA with Tukey's test: ns = not significant, ^*^ *p* < 0.05, ^**^ *p* < 0.01, ^***^ *p* < 0.001, ^****^ *p* < 0.0001.

The mitochondrial ETC is a central driver of tumour progression by sustaining OXPHOS [12], a metabolic adaptation mechanistically linked to the elevated mitoATP levels observed in our single‐organelle analysis (Figure 2B,G). Our observation of elevated mitochondrial Δ*ψ*_m_ further indicates heightened ETC activity in cancer cells (Figure 2D,H). Using the established MitoATP‐nFCM platform, we evaluated three ETC inhibitors: rotenone (complex I), metformin (complex I), and VLX600 (an iron chelator targeting ETC complexes I, II, and IV) (Figure 4A) [38, 39]. Notably, MitoATP‐nFCM analysis demonstrated that rotenone caused significant mitoATP reduction in both malignant (MCF‐7 and MDA‐MB‐231) and normal (MCF‐10A) cells (Figure 4B–E; Figure S14), consistent with its broad toxicity. In contrast, metformin and VLX600 exhibited cancer‐selective effects, minimally affecting normal cells while potently suppressing mitoATP in breast cancer cells (Figure 4C–E). This selectivity likely arises from Δ*ψ*_m_‐dependent drug accumulation in mitochondria [40]. Notably, metformin achieved substantial inhibition at 50 *µ* m, a concentration significantly lower than typical cellular treatment doses (millimolar range) [38]. This implies that targeting mitochondria may broaden metformin's therapeutic window by achieving efficacy at lower concentrations with reduced systemic side effects. VLX600 exhibited superior potency, reducing mitoATP to 52% and 58% of baseline in MCF‐7 and MDA‐MB‐231 cells, respectively (Figure 4D,E). These differential effects were also corroborated in colon cancer models, with VLX600 decreasing mitoATP to 49% in HCT‐15 and 55% in HCT‐116 of controls (Figure 4F–H; Figure S15). Taken together, MitoATP‐nFCM provides an organelle‐resolved platform to evaluate cancer‐targeted ETC inhibitors, a critical step in developing precision therapies with minimized systemic toxicity.

### 2.5 MitoATP‐nFCM Discriminates Cancer‐Selective Inhibitors of Glutamine‐Driven TCA Cycle Metabolism

**FIGURE 5:**
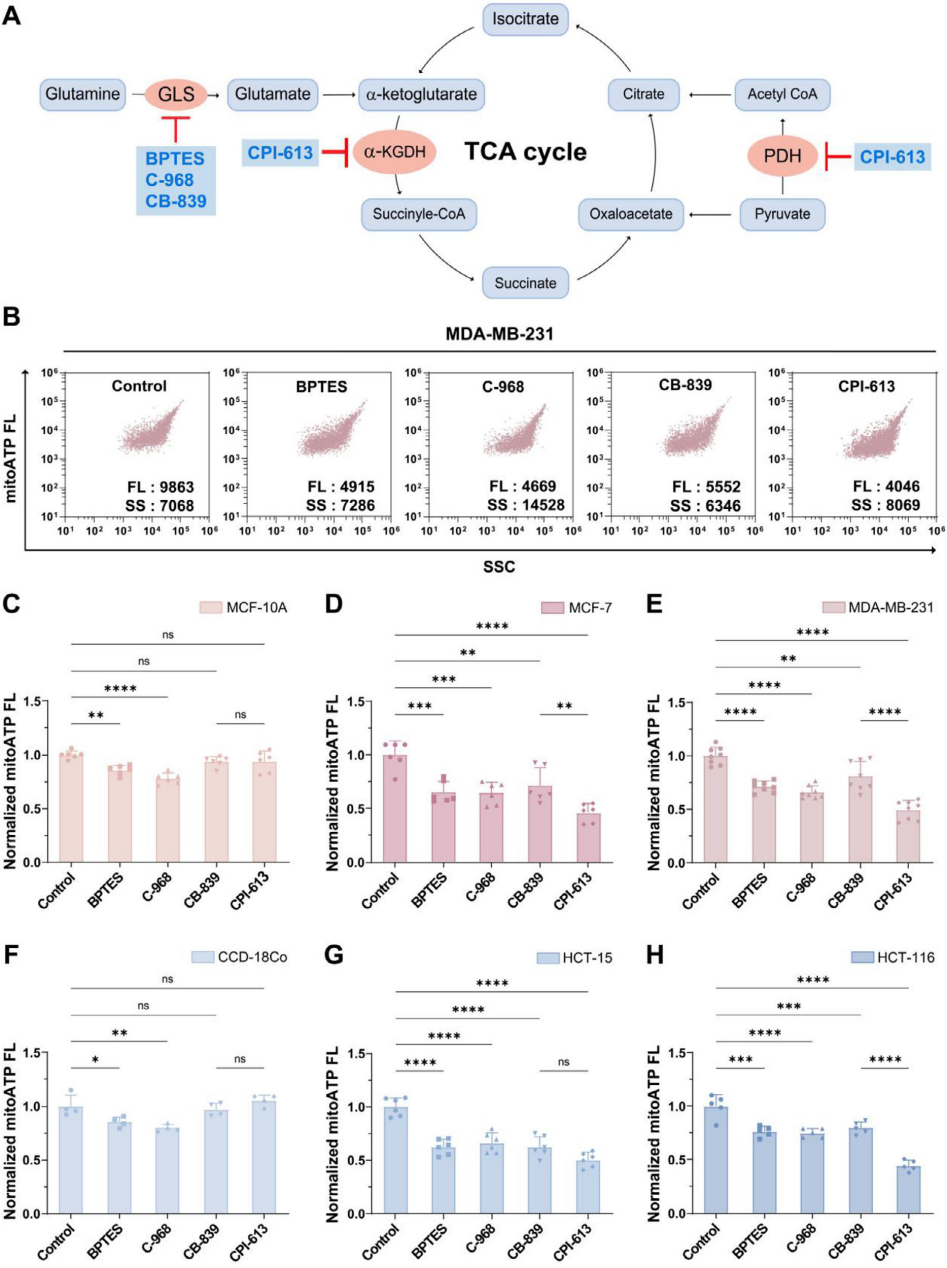
Comparative analysis of mitochondrial tricarboxylic acid (TCA) cycle inhibitors by MitoATP‐nFCM. (A) Metabolic map highlighting intervention targets of TCA cycle inhibitors. (B–E) Isolated mitochondria were subjected to MitoATP‐nFCM analysis following treatment with vehicle control, BPTES, C‐968, CB‐839, or CPI‐613. (B) Bivariate dot‐plots of mitoATP FL burst area versus SSC burst area in MDA‐MB‐231 cells mitochondria. Normalized median mitoATP FL intensity in (C) MCF‐10A (*n* = 6), (D) MCF‐7 (*n* = 6), and (E) MDA‐MB‐231 (*n* = 8) cells mitochondria. (F‐H) Corresponding analysis for colon cell lines: normalized median mitoATP FL intensity in (F) CCD‐18Co (*n* = 4), (G) HCT‐15 (*n* = 6), and (H) HCT‐116 (*n* = 5) cells mitochondria. Error bars represent mean ± SD from ≥ 4 independent experiments. Statistical significance was determined by one‐way ANOVA with Tukey's test: ns = not significant, ^*^ *p* < 0.05, ^**^ *p* < 0.01, ^***^ *p* < 0.001, ^****^ *p* < 0.0001.

A key question in cancer metabolism is how tumors adapt when glycolysis is impaired. Building on our observation of reduced HK2 expression in high‐mitoATP cancer cells (Figure 2F,J), we hypothesized that metabolic rewiring through alternative TCA cycle substrates might compensate for glycolytic defects. In particular, glutamine metabolism may play a key role given its established involvement in OXPHOS‐coupled TCA cycle [33]. The glutaminase (GLS)‐catalyzed conversion of glutamine to glutamate, and the α‐ketoglutarate dehydrogenase (α‐KGDH)‐catalyzed oxidative decarboxylation of α‐ketoglutarate to succinyl‐CoA, represent key regulatory steps in TCA cycle [14, 15]. To test this, we systematically evaluated four inhibitors targeting glutamine‐driven TCA cycle metabolism: two pan‐GLS inhibitors (BPTES and C‐968), a GLS1‐selective inhibitor (CB‐839), and a lipoate analog (CPI‐613) that dually inhibits α‐KGDH and pyruvate dehydrogenase (PDH) (Figure 5A) [41, 42, 43]. At single‐mitochondrion resolution, we uncovered striking divergences: BPTES and C‐968 broadly suppressed mitoATP in both normal (MCF‐10A) and cancerous (MCF‐7 and MDA‐MB‐231) mitochondria, whereas CB‐839 and CPI‐613 showed pronounced cancer selectivity (Figure 5B–E; Figure S16). Notably, CPI‐613 exhibited superior efficacy, reducing mitoATP to 46% in MCF‐7 and 49% in MDA‐MB‐231 cells relative to untreated controls (Figure 5D,E). This cancer‐selective inhibition was replicated in colon models, where CPI‐613 again demonstrated maximal potency, reducing mitoATP to 50% in HCT‐15 and 45% in HCT‐116 cancer cells mitochondria of controls (Figure 5F–H; Figure S17). These findings establish MitoATP‐nFCM as a robust platform for discriminating broad‐spectrum TCA inhibitors (BPTES and C‐968) from cancer‐selective agents (CB‐839 and CPI‐613). The exceptional performance of CPI‐613 likely results from its inhibition of α‐KGDH, which couples the TCA cycle to the ETC [42, 44]. This unique mechanism underscores its therapeutic potential against OXPHOS‐dependent cancers that demonstrate compensatory metabolic plasticity through alternative TCA cycle substrate utilization.

## 3 Conclusion

Our study redefines the therapeutic targeting of OXPHOS‐driven cancers by identifying ​mitochondrial ATP production (not glycolysis) as their metabolic vulnerability node. Through MitoATP‐nFCM development, we achieved three transformative advances: (1) single‐organelle resolution of metabolic heterogeneity between normal and malignant mitochondria, revealing previously inaccessible bioenergetic features of cancer cells that conventional bulk assays could not detect; (2) quantitative validation of cancer cells' heightened vulnerability to mitochondrial metabolic perturbations compared to normal counterparts; and (3) establishment of a robust framework for precision screening of mitochondrial metabolism‐targeted inhibitors, enabling rapid identification of compounds with optimal therapeutic indices (Table S1). These breakthroughs not only establish mitoATP‐depletion as a promising therapeutic strategy for OXPHOS‐dependent cancer cells but also bridge the critical gap between organelle‐level metabolic phenotyping and targeted drug discovery. The platform's unique ability to discriminate cancer‐selective inhibitors (e.g., BDQ, VLX600, CPI‐613) from broad‐spectrum agents provides both a mechanistic blueprint for understanding mitochondrial metabolic dependencies in cancer and a practical roadmap for developing next‐generation therapies with enhanced tumor specificity and reduced systemic toxicity. Importantly, this approach may be extended beyond cancer to other pathologies involving mitochondrial dysfunction, positioning MitoATP‐nFCM as a versatile platform for advancing precision medicine across multiple disease areas.

## 4 Experimental Section

### 4.1 Materials and Reagents

BioTracker ATP‐Red 1 (SCT045) was purchased from Merck‐Millipore. Hoechst 33342 (C1028) and Mito‐Tracker green (C1048) were obtained from Beyotime. Lyso‐Tracker Green DND‐26 (MB6042) was sourced from Meilunbio. Carbonyl cyanide m‐chlorophenyl hydrazone (CCCP, HY‐100941), oligomycin A (OligA, HY‐16589), bedaquiline (BDQ, HY‐14881), rotenone (HY‐B1756), metformin (HY‐17471), VLX600 (HY‐12406), BPTES (HY‐12683), C‐968 (HY‐12682), CB‐839 (HY‐11248), and CPI‐613 (HY‐15453) were acquired from MedChemExpress. 5’‐Nucleotides & Nucleosides (K10025A), paraformaldehyde (30525‐89‐4), digitonin (11024‐24‐1), and DiOC_6_(3) (53213‐82‐4) were obtained from Sigma‐Aldrich. The primary anti‐ATP5C1 antibody (10910‐1‐AP, Proteintech) was used for ATP synthase detection, along with a secondary CoraLite 488‐conjugated goat anti‐mouse IgG(H+L) antibody (SA00013‐1, Proteintech). The anti‐hexokinase 2 antibody (ab104836) was obtained from Abcam. Mitochondrial buffer (MT buffer) was prepared with 250 mm sucrose, 10 mm HEPES, 1 mm EGTA, 4.2 mm sodium succinate hexahydrate, and 1 mm potassium dihydrogen phosphate (pH adjusted to 7.4 using 1 m KOH) and served as a negative control, as well as for mitochondria storage, washing, and staining. Differential centrifugation was performed using a Centrifuge 5424 R (Eppendorf), and ultrapure water from a Milli‐Q RG unit (Millipore) was used throughout the experiments.

### 4.2 Cell Culture

Human normal mammary immortalized epithelial cells (MCF‐10A, RRID: CVCL_0598) was obtained from American Type Culture Collection (ATCC, CRL‐10317) in December 2021. Hormone receptor‐positive breast cancer cells (MCF‐7, RRID: CVCL_0031) was obtained from ATCC (HTB‐22) in October 2020. Triple‐negative breast cancer cells (MDA‐MB‐231, RRID: CVCL_0062) was obtained from the ATCC (HTB‐26) in January 2020. Normal colon fibroblast cells (CCD‐18Co, RRID: CVCL_2379) was obtained from the ATCC (CRL‐1459) in May 2016. Human colorectal carcinoma (HCT‐15, RRID: CVCL_0292; HCT‐116, RRID: CVCL_0291) were obtained from the ATCC (HCT‐15, CCL‐225; HCT‐116, CCL‐247) in October 2015. All cell lines have been authenticated using STR profiling. All experiments were performed with mycoplasma‐free cells. MCF‐10A cells were cultured in MCF‐10A‐specific medium (Procell), while MCF‐7, MDA‐MB‐231, and CCD‐18Co cells were cultured in Dulbecco's Modified Eagle Medium (DMEM, Hyclone). HCT‐15 cells were grown in RPMI‐1640 medium (Hyclone), and HCT‐116 cells were propagated in McCoy's 5A medium (Procell). All media were supplemented with 10% fetal bovine serum (FBS, ExCell Bio) and penicillin‐streptomycin (Hyclone). Cells were incubated at 37°C in a humidified 5% (v/v) CO_2_ atmosphere.

### 4.3 Measurement of Photophysical Properties of ATP‐Red 1

Absorption and fluorescence spectra were acquired using a SpectraMax iD5 Multi‐Mode Microplate Reader (Molecular Device) with excitation and emission slit widths set to 1 nm. The ATP‐Red 1 probe (10 mm) was prepared in dimethyl sulfoxide (DMSO) and stored at −20°C until use. Spectra were recorded immediately after adding purified mitochondria or adenine nucleotides.

### 4.4 Scanning Confocal Microscopy Analysis

Cells were seeded on 35‐mm confocal dishes (Biosharp). For intracellular localization studies, cells were sequentially stained with ATP‐Red 1 (10 *µ* m, 30 min), followed by Mito‐Tracker Green (0.2 *µ* m, 15 min) or Lyso‐Tracker Green DND‐26 (0.2 *µ* m, 15 min), and finally Hoechst 33342 (15 min) at 37°C. After three Phosphate Buffered Saline (PBS) washes, images were acquired using a Leica SP8 laser scanning confocal microscope equipped with a 63× oil‐immersion objective and analyzed using LAS X Life Science Microscope Software (Leica).

### 4.5 Mitochondria Isolation

Cells were cultured in 150‐mm Falcon dishes with media replenishment every 2 days. At 90%–95% confluence (∼2 × 10^7^ cells/dish), cells were harvested in ice‐cold PBS, and centrifuged (950 × g, 4°C, 5 min) to remove debris. Mitochondria were isolated using a Mitochondria Isolation Kit (Thermo Fisher) according to the manufacturer's protocol. Briefly, cell pellets were resuspended in 1 mL reagent A for about 2 min, homogenized with 60 strokes in a Dounce tissue grinder (Wheaton) on ice, then mixed with 1 mL cold reagent C. After centrifugation (700 × g, 4°C, 10 min) to remove nuclei and cell debris, the supernatant was sequentially centrifuged (3 000 × g, 4°C, 15 min; then 12 000 × g, 4°C, 5 min) to pellet mitochondria. The final mitochondrial pellet was resuspended in 200 *µ*L of ice‐cold MT buffer and placed on ice before use. The protein concentration of purified mitochondria was determined by UV absorbance at 280 nm using a SpectraMax QuickDrop spectrophotometer (Molecular Device).

### 4.6 Drug Treatment and Fluorescent Staining of Isolated Mitochondria

Purified mitochondria (20 *µ*g protein) were treated with various inhibitors at indicated concentrations in 200 *µ*L MT buffer (2 h, 37°C), with 0.5% DMSO as vehicle control. Post‐treatment, mitochondria were pelleted (12 000 × g, 4°C, 5 min) and washed once with MT buffer. For mitochondrial ATP staining, mitochondria were resuspended in 200 *µ*L MT buffer containing ATP‐Red 1 (specified concentrations) and incubated for 30 min at 37°C. Mitochondrial membrane potential was assessed as previously described [19]. Briefly, mitochondria were stained with 1 mm DiOC_6_(3) in 200 *µ*L MT buffer for 30 min at 37°C. After staining, mitochondria were centrifuged (12 000 × g, 4°C, 5 min) to remove unbound dye, washed once with 200 *µ*L of MT buffer, and then resuspended with 100 *µ*L of MT buffer on ice for nano‐flow cytometry analysis.

### 4.7 Immunofluorescence Labeling of Isolated Mitochondria

For inner mitochondrial membrane ATP synthase protein labeling, mitochondria (30 *µ*g) were mixed with 1% paraformaldehyde in 200 *µ*L MT buffer for 15 min at room temperature, pelleted (12 000 × g, 4°C, 5 min), then permeabilized in 100 *µ*L incubation buffer (250 *µ*g/mL digitonin in MT buffer) containing 0.5 *µ*g anti‐ATP5C1 antibody and incubated overnight at room temperature with shaking. For outer mitochondrial membrane hexokinase 2 protein labeling, mitochondria (20 *µ*g) were similarly fixed and incubated overnight with 0.5 *µ*g anti‐HK2 antibody in 100 *µ*L MT buffer. After primary antibody incubation, samples were washed twice with 100 *µ*L MT buffer (12 000 × g, 4°C, 5 min), then incubated with 1 *µ*g CoraLite 488‐conjugated Goat anti mouse IgG(H+L) in 100 *µ*L MT buffer at room temperature with shaking for 2 h. Finally, mitochondria were washed twice with 100 *µ*L of MT buffer (12 000 × g, 4°C, 5 min) and resuspended in 100 *µ*L of MT buffer for nano‐flow cytometry analysis.

### 4.8 Nano‐Flow Cytometry (nFCM) Analysis

Mitochondrial analysis was performed using a laboratory‐built nFCM system equipped with a 488‐nm continuous‐wave solid‐state laser (Newport) and three detection channels (side scatter and two fluorescence channels), following established methodology [18]. Photon bursts were simultaneously recorded from: (i) the side scatter (SSC) channel (PMT, R3788, Hamamatsu) and (ii) two fluorescence channels (520/35 and 630/69 nm bandpass filters) each detected by separate PMTs (R928, Hamamatsu). Data acquisition and processing were performed using a custom LabVIEW program.

### 4.9 Conventional Flow Cytometry Analysis

For cellular ATP measurement, cells (∼5 × 10^6^ particles/mL) were stained with ATP‐Red 1 (10 *µ* m, 30 min) at 37°C, washed with PBS, and analyzed using a BD FACS Aria II flow cytometer (Becton Dickinson). Fluorescence intensities were quantified as median values using FlowJo software (v10.8.1), with data normalized to baseline cellular levels.

### 4.10 Statistical Analysis

Data analysis was performed using GraphPad Prism 9.0, Origin 9.0, and FlowJo software. Results were represented as mean ± standard deviation (SD) from ≥ 3 independent experiments. Statistical significance was determined by unpaired two‐tailed Student's t‐test or one‐way ANOVA with Tukey's test for multiple comparisons. Significance levels are denoted as follows: ns = not significant, ^*^ *p* < 0.05, ^**^ *p* < 0.01, ^***^ *p* < 0.001, and ^****^ *p* < 0.0001 (95% confidence level).

## Author Contributions

X. Xiao and X. Yan conceived and designed the study. X. Xiao and C. Lu performed the experiments and analyzed data. H. Chen and J. Zhou assisted with data acquisition. Y. Hu and H. Di contributed to methodology development and validation. G. Su and X. Yan supervised the project and secured funding. X. Xiao drafted the manuscript with critical revisions by X. Xiao, G. Su, and X. Yan. All authors reviewed and approved the final manuscript.

## Conflicts of Interest

X. Yan declares competing financial interest as a cofounder of NanoFCM Inc., a company committed to commercializing the nano‐flow cytometry (nFCM) technology.

## Supporting information




**Supporting File**: advs74253‐sup‐0001‐SuppMat.pdf.

## Data Availability

The data that support the findings of this study are available in the supplementary material of this article.
