# Supplementary material for: Single‐Mitochondrion ATP Profiling Directs Discovery of Targetable OXPHOS Dependency in Cancers
Source: Adv Sci (Weinh). 2026 Feb 13;13(21):e13341. doi: 10.1002/advs.202513341 (PMC13073232; doi:10.1002/advs.202513341)
Supplement: Supplementary file 1 — Supporting File: advs74253‐sup‐0001‐SuppMat.pdf. [file ADVS-13-e13341-s001.pdf]

Supporting Information

Single-Mitochondrion ATP Profiling Directs Discovery of Targetable OXPHOS Dependency in Cancers

Xu Xiao[1], Cheng Lu[1], Hao Chen[1], Jing Zhou[1], Yunyun Hu[1], Haonan Di[1], Guoqiang Su,[2,*] and Xiaomei Yan[1,*]

[1]Department of Chemical Biology, MOE Key Laboratory of Spectrochemical Analysis & Instrumentation, Fujian Key Laboratory of Chemical Biology (Xiamen University), State Key Laboratory of Physical Chemistry of Solid Surfaces, Collaborative Innovation Center of Chemistry for Energy Materials, College of Chemistry and Chemical Engineering, Xiamen University, Xiamen, Fujian 361005, China

E-mail: xmyan@xmu.edu.cn

[2]Department of Colorectal Tumor Surgery, Xiamen Key Laboratory of Early Cancer Diagnosis and Treatment, The First Affiliated Hospital of Xiamen University, School of Medicine, Xiamen University, Xiamen, Fujian 361003, China

E-mail: suguoqiang@xmu.edu.cn

* To whom correspondence should be addressed. E-mail: xmyan@xmu.edu.cn, suguoqiang@xmu.edu.cn

Figure S1

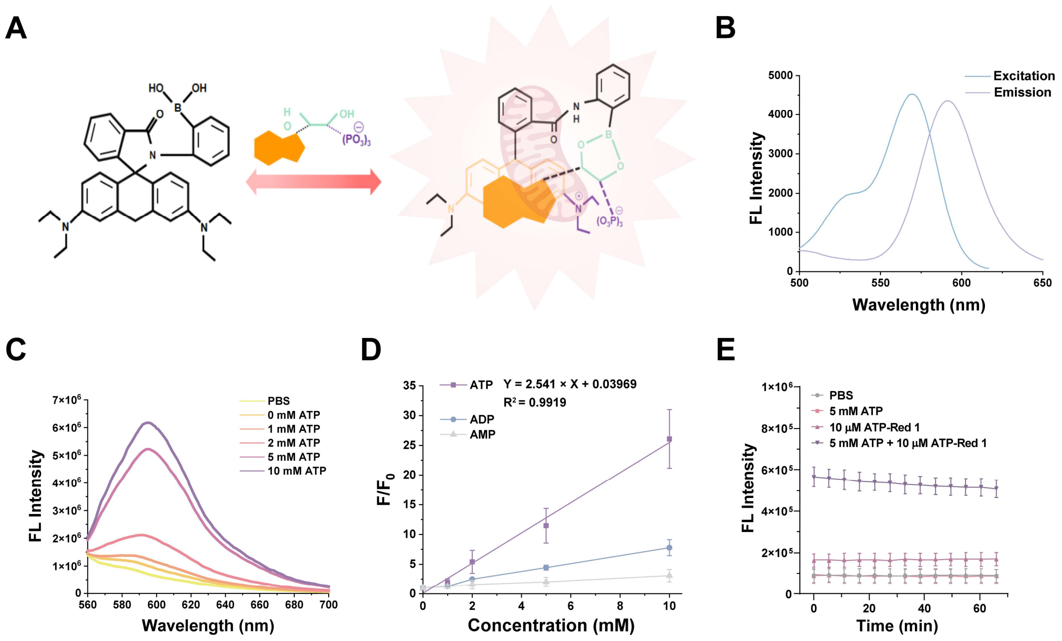

Figure S1. Characterization of ATP-Red 1 as a mitochondrial ATP (mitoATP)-specific fluorescent probe. (A) Molecular mechanism of ATP-Red 1 activation upon ATP binding, showing structural rearrangement that triggers fluorescence. (B) Fluorescence excitation and emission spectra of ATP-Red 1 (10 μM) in the presence of isolated mitochondria (10 μg mitochondrial protein). (C) Fluorescence emission spectra of ATP-Red 1 (10 μM) titrated with 0-10 mM ATP. (D) Relative fluorescence intensities (F/F_0 at 590 nm) of ATP-Red 1 (10 μM) in response to ATP (purple), ADP (blue), and AMP (gray) across 0-10 mM concentrations ($n = 4$). F_0 represents baseline fluorescence in PBS (pH 7.4). (E) Stability assessment of ATP-Red 1. The fluorescence intensity of ATP-Red 1 (10 μM) in the presence of 5 mM ATP was monitored over 60 min after a 2-hour pre-incubation, and compared with relevant controls (PBS, ATP alone, and probe alone) ($n = 8$). Fluorescence measurements were obtained at $\lambda_{ex} = 488$ nm. Data represent mean ± SD from at least 4 independent experiments.

Figure S2

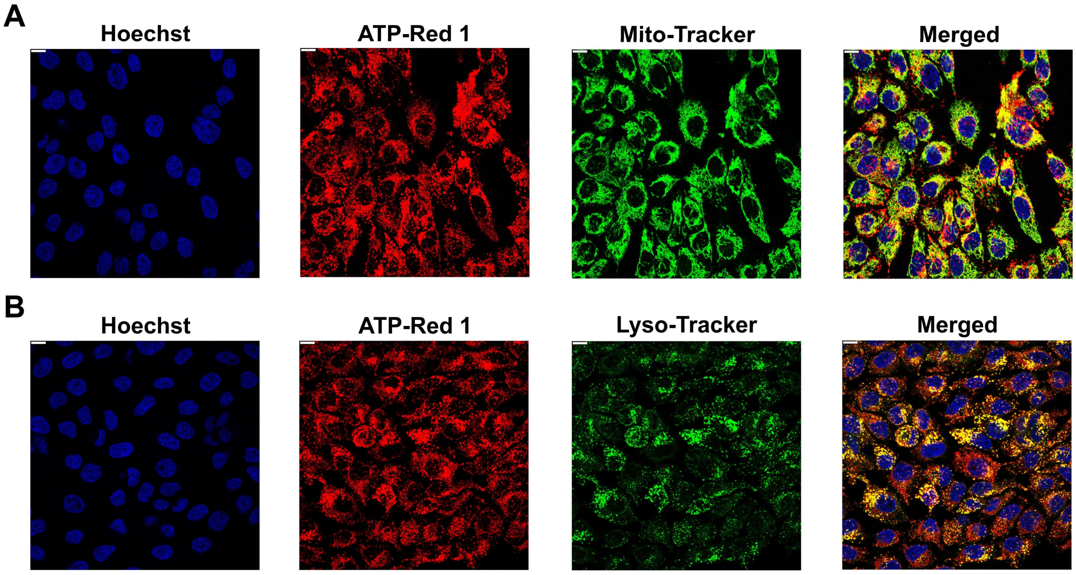

Figure S2. Subcellular localization analysis of ATP-Red 1 in MCF-7 cells by confocal microscopy. (A) Mitochondrial colocalization: Cells were sequentially stained with ATP-Red 1 (10 μM, 37°C, 30 min; red channel), Mito-Tracker Green (0.2 μM, 37°C, 15 min; green channel), and Hoechst 33342 (37°C, 15 min; blue channel, nuclei). (B) Lysosomal colocalization: Cells were similarly processed with Lyso-Tracker Green (0.2 μM, 37°C, 15 min; green channel) instead of Mito-Tracker Green. Yellow: Colocalized signals in merged images. Scale bar: 10 μm.

Figure S3

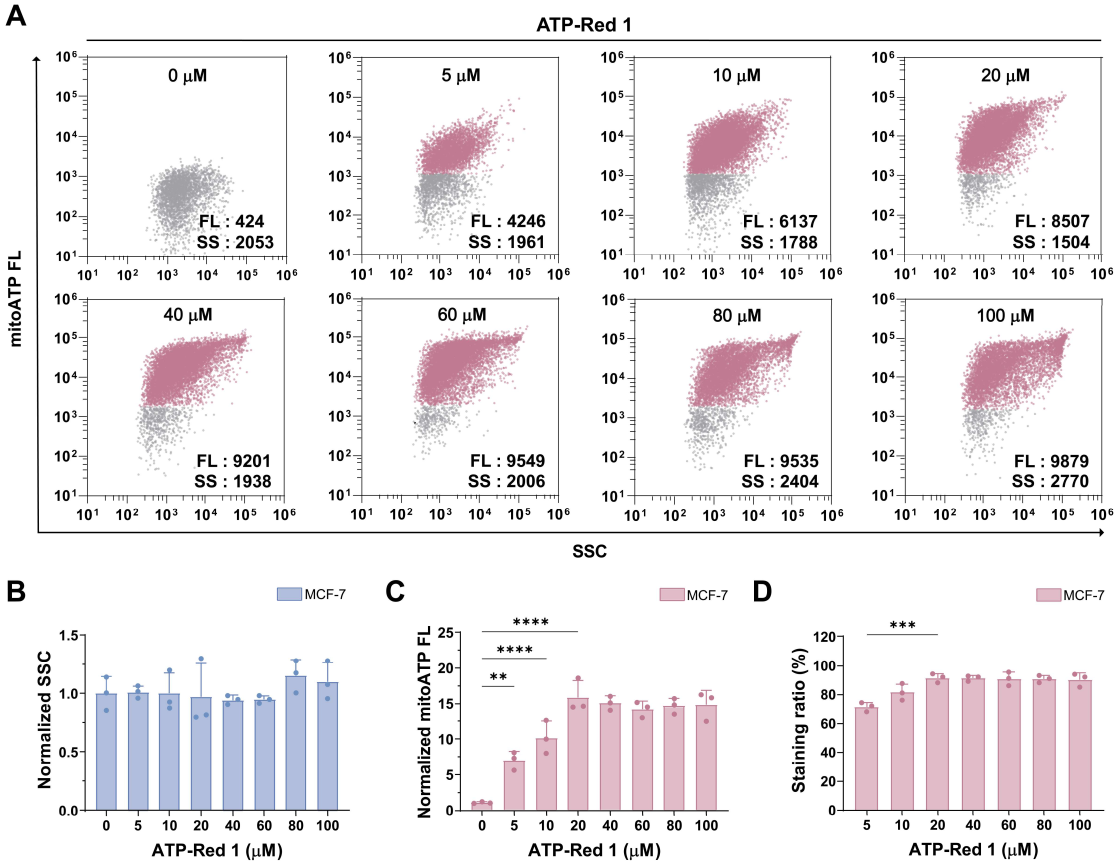

Figure S3. Quantitative analysis of ATP-Red 1 staining in isolated mitochondria using nFCM. (A-D) Mitochondria isolated from MCF-7 breast cancer cells were stained with increasing ATP-Red 1 concentrations (0-100 μM) at 37°C for 30 min and then analyzed by nFCM. (A) Bivariate dot-plots showing mitoATP fluorescence (FL) versus side scatter (SSC) burst areas were acquired over 60 s per sample. Numerical values indicate median FL and SSC intensities for each condition. (B-D) Quantitative parameters derived from nFCM analysis: (B) Normalized median SSC intensity, (C) normalized median FL intensity, and (D) percentage of ATP-Red 1-positive mitochondria (staining ratio). Data represent mean ± SD from 3 independent experiments. Statistical significance (unpaired two-tailed Student's t-test) is denoted: $^{**}p < 0.01$, $^{***}p < 0.001$, $^{****}p < 0.0001$.

Figure S4

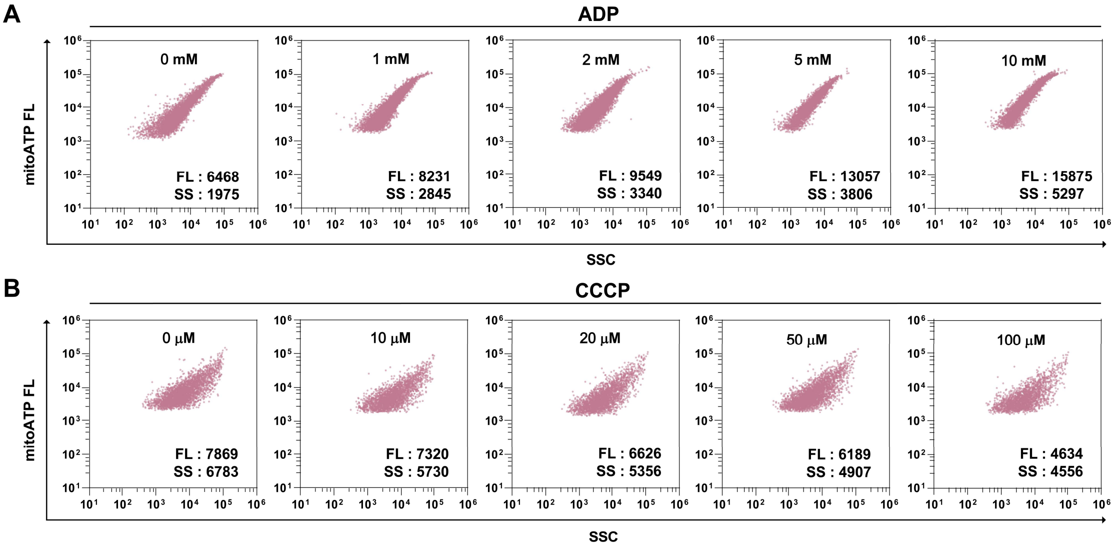

Figure S4. Dose-dependent effects of ADP and CCCP on mitochondrial ATP levels analyzed by MitoATP-nFCM. (A) Bivariate dot-plots of mitoATP FL burst area versus SSC burst area of isolated mitochondria treated with ADP (0-10 mM) at 37°C for 2 h. (B) Bivariate dot-plots of mitoATP FL burst area versus SSC burst area for mitochondria treated with CCCP (0-100 μM) at 37°C for 2 h. Numerical values indicate median FL and SSC intensities for each concentration.

Figure S5

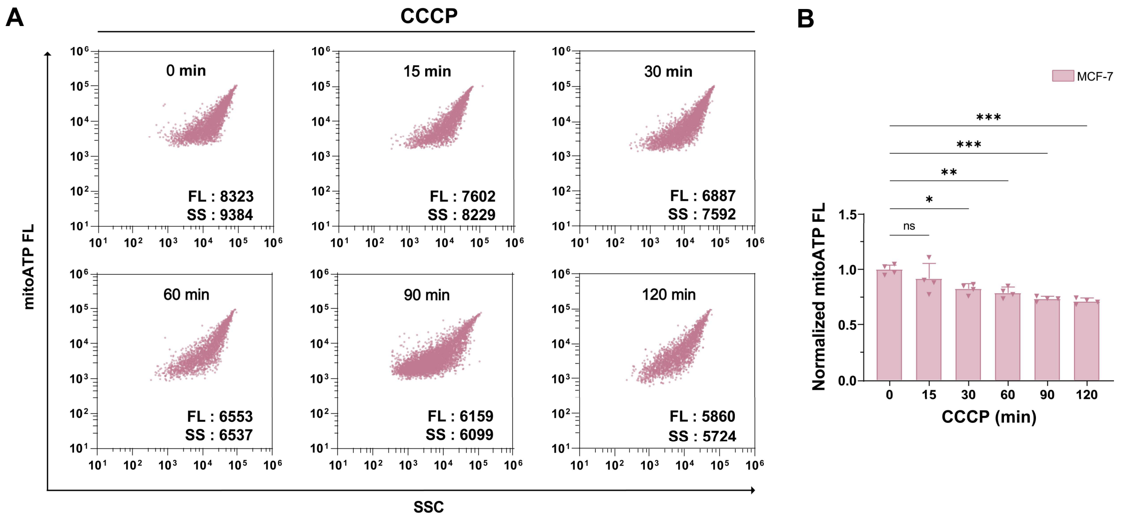

Figure S5. Time-dependent effects of CCCP on mitochondrial ATP levels analyzed by MitoATP-nFCM. (A,B) Mitochondria isolated from MCF-7 cells were treated with CCCP (100 μM) at the varying times from 0-120 min, stained with ATP-Red 1, and then analyzed by nFCM. (A) Bivariate dot-plots of mitoATP FL burst area versus SSC burst area were derived from 60 s of data. (B) Normalized median FL intensity showing progressive ATP depletion with CCCP exposure time. Data represent mean ± SD from 4 independent experiments. Statistical significance (one-way ANOVA with Tukey's test) is denoted: ns = not significant, *$p < 0.05$, **$p < 0.01$, ***$p < 0.001$.

Figure S6

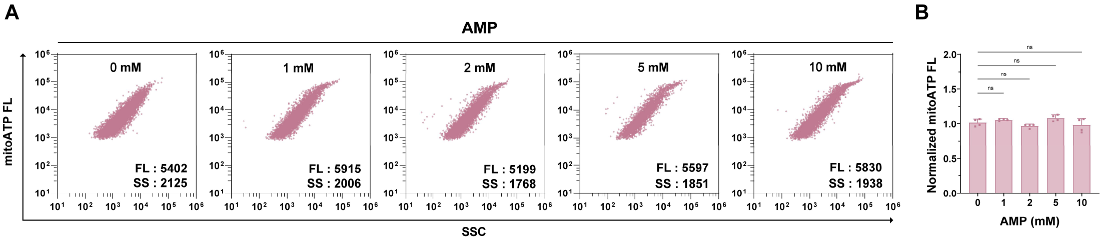

Figure S6. Dose-dependent effects of AMP on mitochondrial ATP levels analyzed by MitoATP-nFCM. (A) Bivariate dot-plots of mitoATP FL burst area versus SSC burst area of isolated mitochondria treated with AMP (0-10 mM) at 37°C for 2 h. (B) Quantitative analysis of normalized median mitoATP FL intensity for AMP (0-10 mM). Data represent mean ± SD from 4 independent experiments. Statistical significance (one-way ANOVA with Tukey's test) is denoted: ns = not significant.

Figure S7

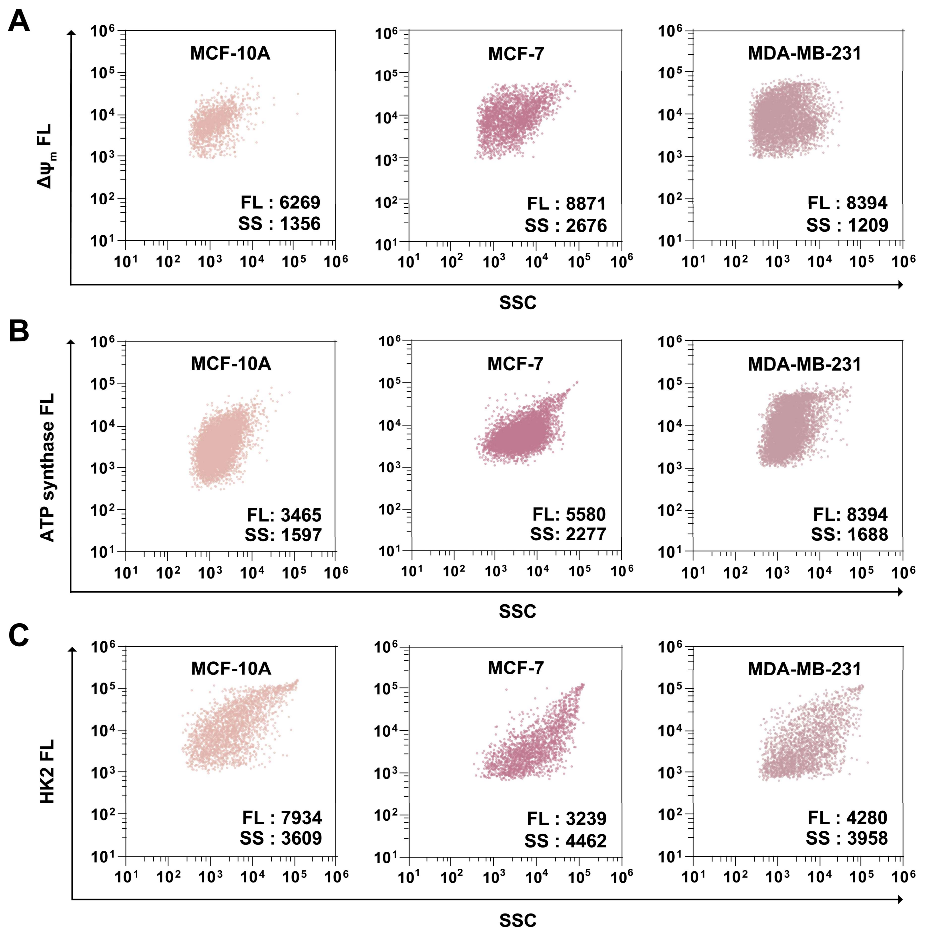

Figure S7. Multiparameter characterization of isolated mitochondria from breast cell lines by nFCM. (A) Bivariate dot-plots of mitochondrial membrane potential ($\Delta\psi_m$) FL burst area versus SSC burst area for mitochondria isolated from three cell lines: MCF-10A, MCF-7, and MDA-MB-231. (B) Mitochondrial ATP synthase protein expression levels. (C) Hexokinase 2 (HK2) protein expression levels. All data represent isolated mitochondria analyzed over 60 s per sample, with annotated median FL/SSC intensities.

Figure S8

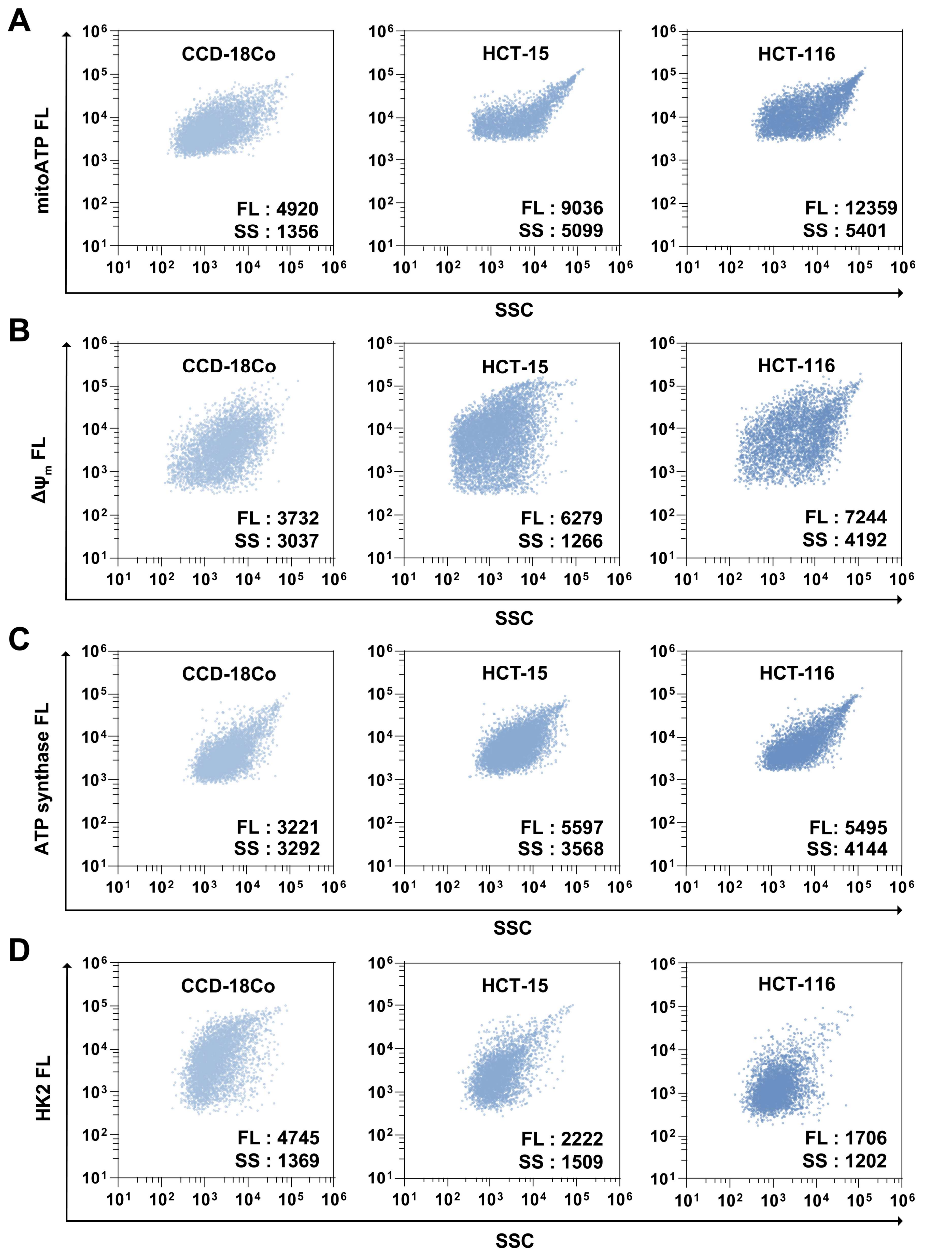

Figure S8. Multiparameter characterization of isolated mitochondria from colon cell lines by nFCM. (A) Bivariate dot-plots of mitoATP FL burst area versus SSC burst area for mitochondria isolated from three colon cell lines: CCD-18Co, HCT-15 and HCT-116. (B) Mitochondrial membrane potential ($\Delta\psi_m$) measurements. (C) Mitochondrial ATP synthase protein expression levels. (D) Mitochondrial HK2 protein expression levels. All data represent isolated mitochondria analyzed over 60 s per sample, with annotated median FL/SSC intensities.

Figure S9

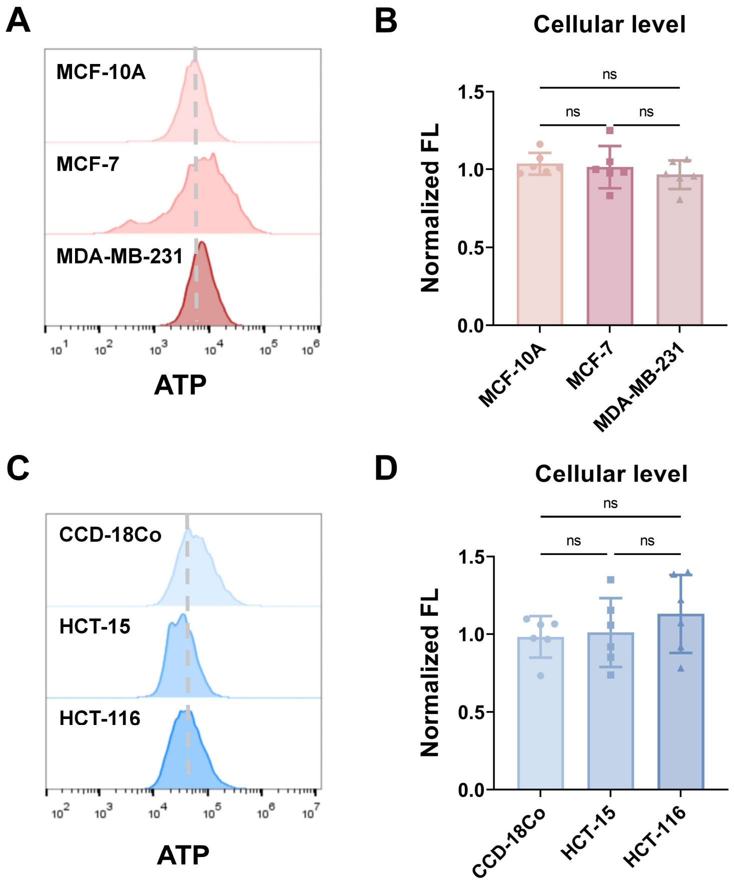

Figure S9. Flow cytometric analysis of cellular ATP levels between normal and cancer cell lines. (A-D) Cells were stained with ATP-Red 1 (10 μM) at 37°C for 30 min and then analyzed by flow cytometry. (A) Distribution histograms of ATP-Red 1 FL intensity for breast cell lines: MCF-10A, MCF-7, and MDA-MB-231. (B) Bar graph of the median fluorescence of ATP-Red 1 for three breast cell lines ($n = 6$). (C) Distribution histograms of ATP-Red 1 FL intensity for colon cell lines: CCD-18Co, HCT-15, and HCT-116. (D) Bar graph of the median fluorescence of ATP-Red 1 for three colon cell lines ($n = 6$). Data represent mean ± SD from 6 independent experiments. Statistical significance (one-way ANOVA with Tukey's test) is denoted: ns = not significant.

Figure S10

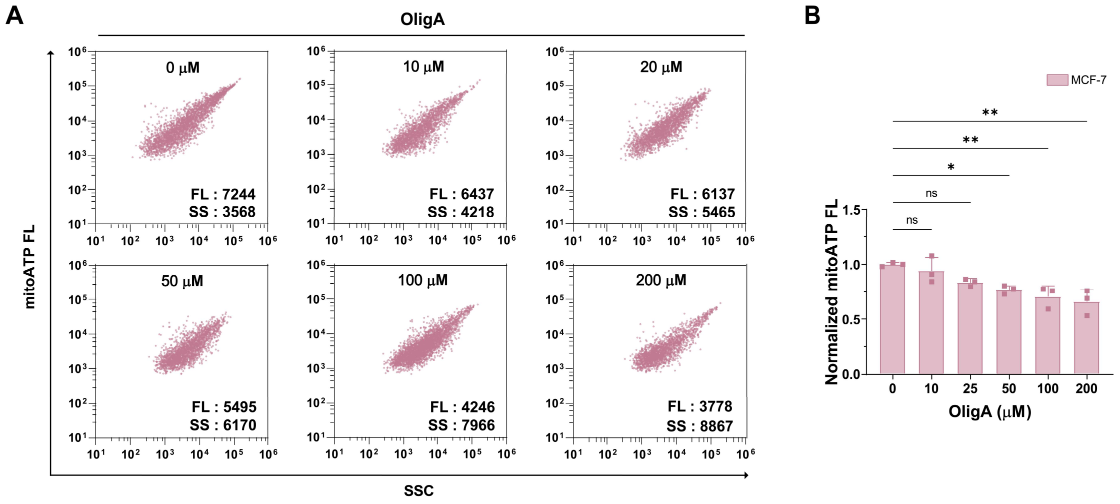

Figure S10. Dose-dependent effects of oligomycin A (OligA) on mitochondrial ATP assessed by MitoATP-nFCM. (A,B) Mitochondria isolated from MCF-7 cells were treated with OligA at the increasing concentrations from 0-200 μM at 37°C for 2 h as determined by MitoATP-nFCM analysis. (A) Bivariate dot-plots of mitoATP FL burst area versus SSC were derived from 60 s of data. (B) Quantification of normalized mitoATP median FL intensity. Data represent mean ± SD from 3 independent experiments. Statistical significance (one-way ANOVA with Tukey's test) is denoted: ns = not significant, *p < 0.05, **p < 0.01.

Figure S11

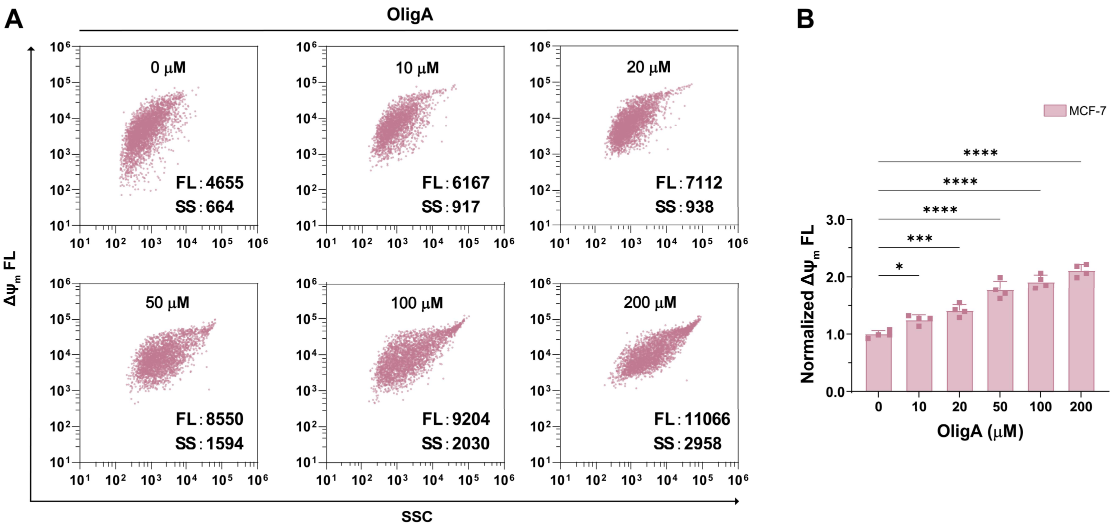

Figure S11. Dose-dependent effects of OligA on mitochondrial membrane potential ($\Delta\psi_m$) assessed by nFCM. (A,B) Mitochondria isolated from MCF-7 cells were treated with OligA at the increasing concentrations from 0-200 μM at 37°C for 2 h before $DiOC_6(3)$ staining and then analyzed by nFCM. (A) Bivariate dot-plots of mitochondrial $\Delta\psi_m$ FL burst area versus side scatter burst area were derived from 60 s of data. (B) Quantification of normalized $\Delta\psi_m$ median FL intensity. Data represent mean ± SD from 4 independent experiments. Statistical significance (one-way ANOVA with Tukey's test) is denoted: *$p < 0.05$, ***$p < 0.001$, ****$p < 0.0001$.

Figure S12

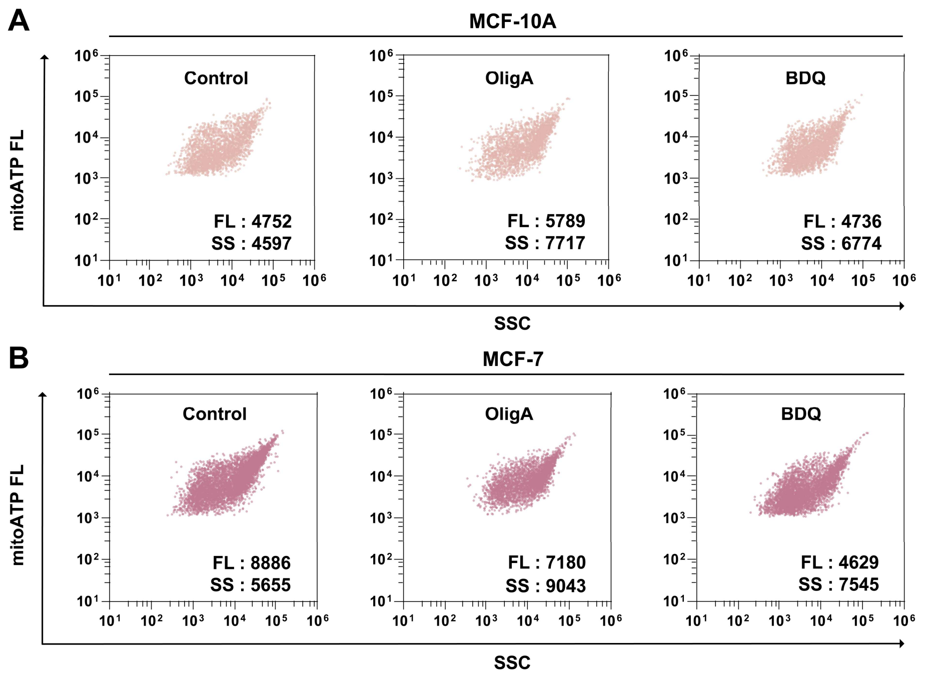

Figure S12. Comparing the effects of ATP synthase inhibitors on mitoATP levels in normal versus cancerous breast cells by MitoATP-nFCM. (A,B) Bivariate dot-plots of mitoATP FL burst area versus SSC burst area for mitochondria isolated from (A) MCF-10A and (B) MCF-7 cells treated with vehicle control (0.5% DMSO, Control), oligomycin A (OligA, 50 μM), or bedaquiline (BDQ, 50 μM) at 37°C for 2 h.

Figure S13

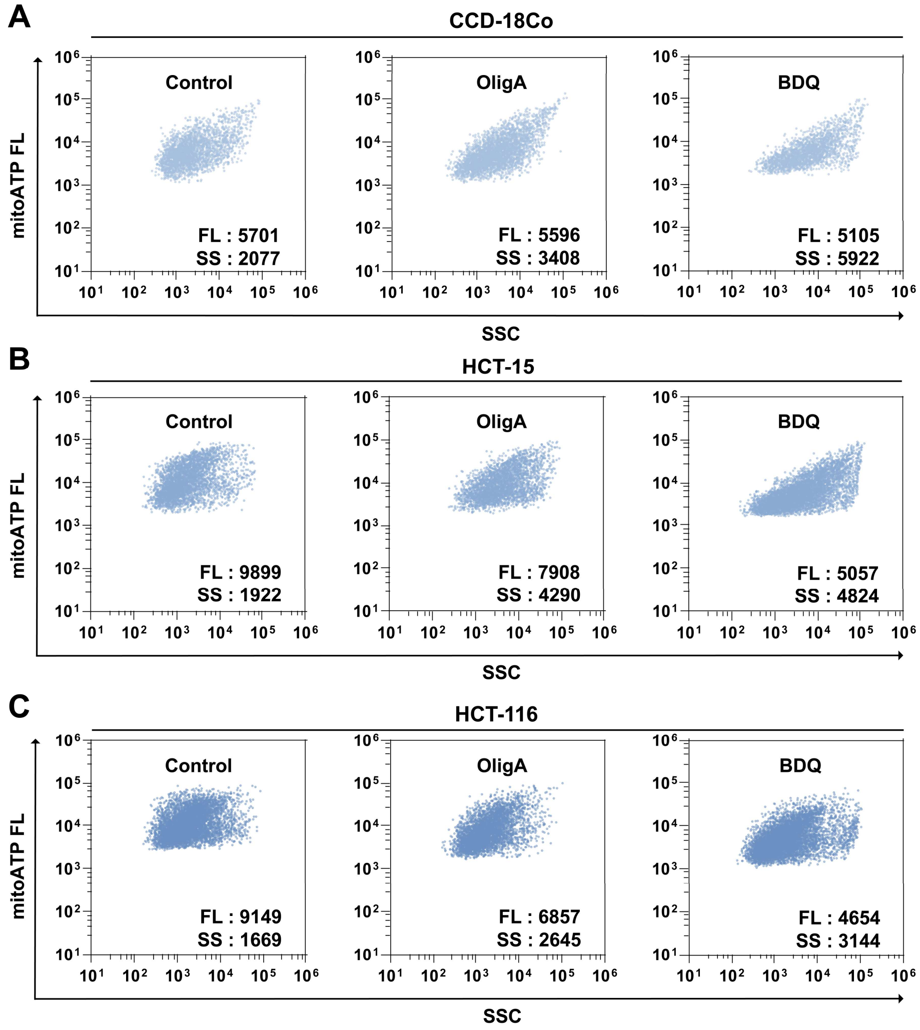

Figure S13. Comparing the effects of ATP synthase inhibitors on mitoATP levels in normal versus malignant colorectal cells by MitoATP-nFCM. (A-C) Bivariate dot-plots of mitoATP FL burst area versus SSC burst area for mitochondria isolated from (A) CCD-18Co, (B) HCT-15, (C) HCT-116 cells treated with (0.5% DMSO, Control), oligomycin A (OligA, 50 μM), or bedaquiline (BDQ, 50 μM) at 37°C for 2 h.

Figure S14

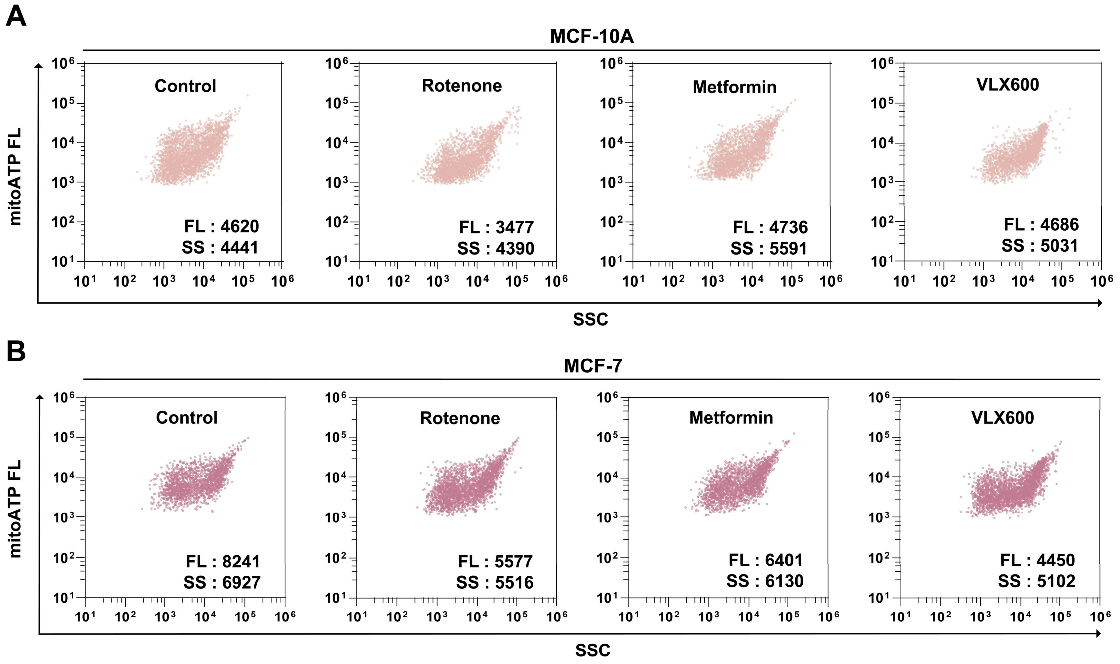

Figure S14. Comparing the effects of electron transport chain (ETC) inhibitors on mitoATP levels in normal versus malignant colorectal cells by MitoATP-nFCM. (A,B) Bivariate dot-plots of mitoATP FL burst area versus SSC burst area for mitochondria isolated from (A) MCF-10A and (B) MCF-7 cells treated with vehicle control (0.5% DMSO, Control), rotenone (50 µM), metformin (50 µM), or VLX600 (50 µM) at 37°C for 2 h.

Figure S15

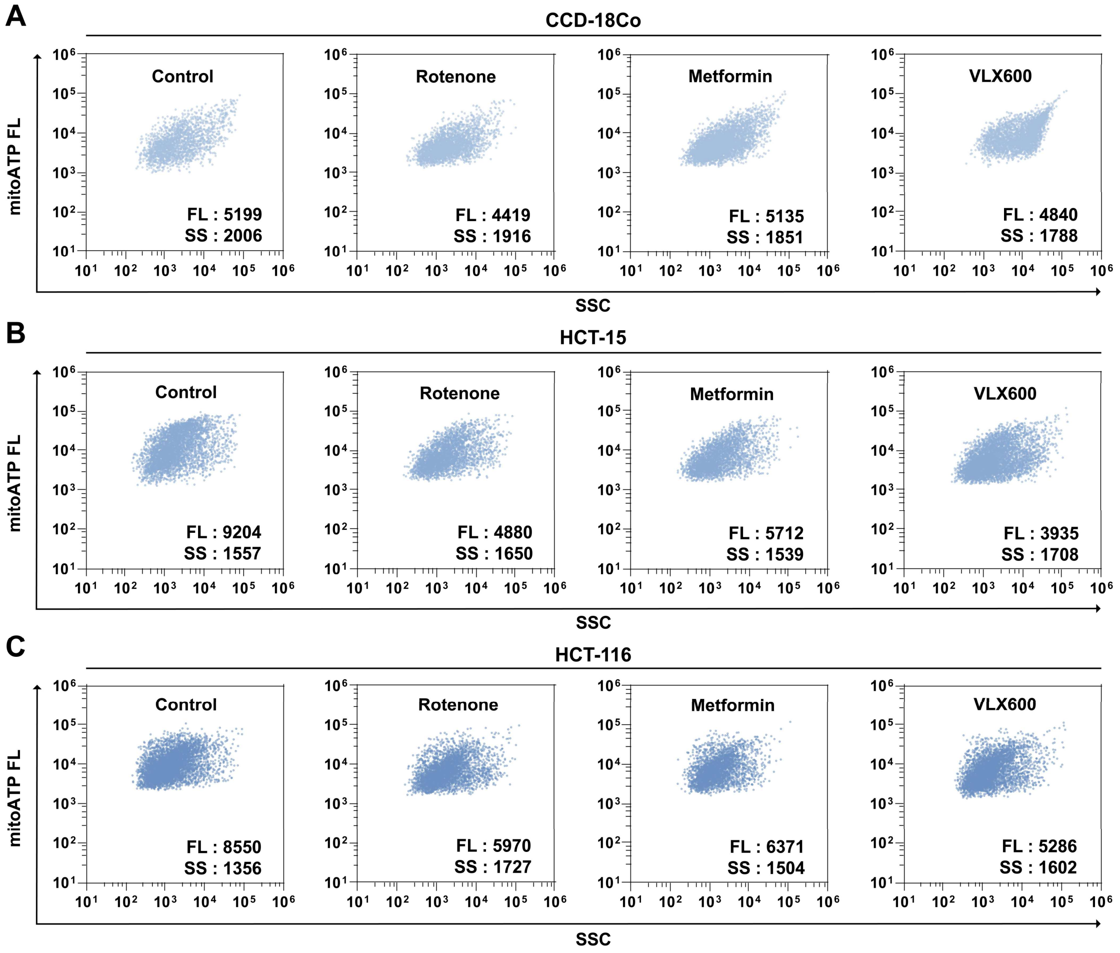

Figure S15. Comparing the effects of ETC inhibitors on mitoATP levels in normal versus malignant colorectal cells by MitoATP-nFCM. (A-C) Bivariate dot-plots of mitoATP FL burst area versus SSC burst area for mitochondria isolated from (A) CCD-18Co, (B) HCT-15, (C) HCT-116 cells treated with vehicle control (0.5% DMSO, Control), rotenone (50 μM), metformin (50 μM), or VLX600 (50 μM) at 37°C for 2 h.

2.15 Figure S16

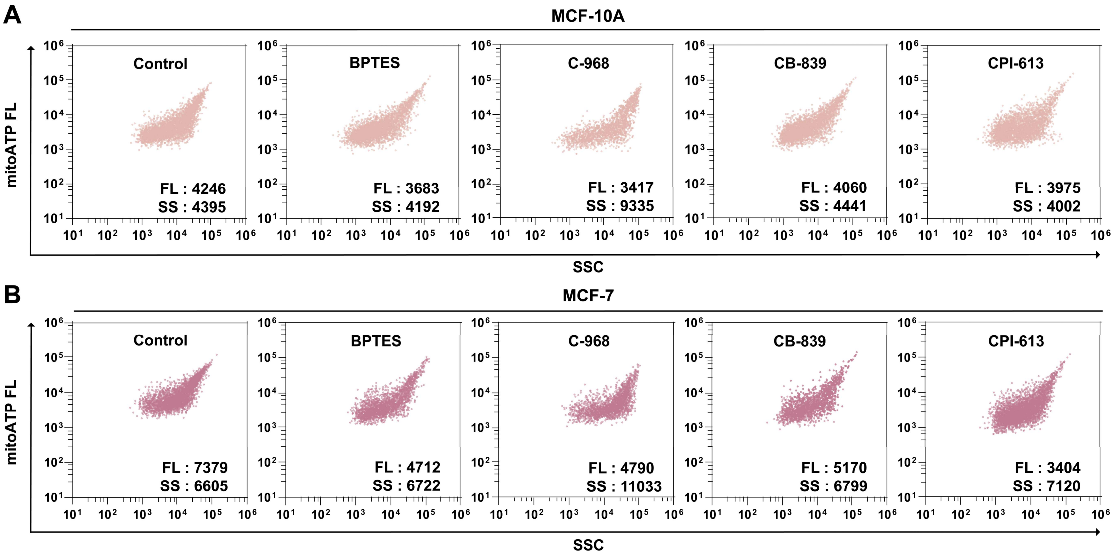

Figure S16. Comparing the effects of tricarboxylic acid (TCA) cycle inhibitors on mitoATP levels in normal versus malignant colorectal cells by MitoATP-nFCM. (A,B) Bivariate dot-plots of mitoATP FL burst area versus SSC burst area for mitochondria isolated from (A) MCF-10A and (B) MCF-7 cells treated with vehicle control (0.5% DMSO, Control), BPTES, C-968, CB-839, or CPI-613 at 37°C for 2 h. The inhibitor concentrations were all at 50 μM.

Figure S17

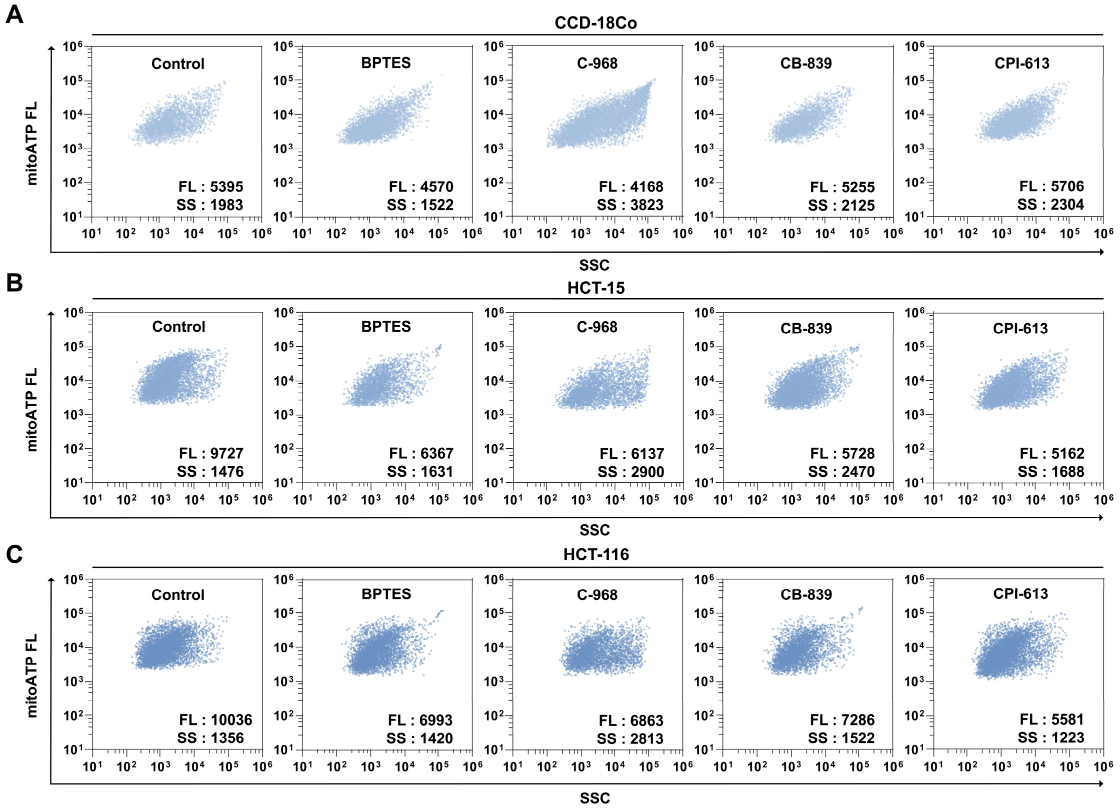

Figure S17. Comparing the effects of TCA cycle inhibitors on mitoATP levels in normal versus malignant colorectal cells by MitoATP-nFCM. (A-C) Bivariate dot-plots of mitoATP FL burst area versus SSC burst area for mitochondria isolated from (A) CCD-18Co, (B) HCT-15, (C) HCT-116 cells treated with vehicle control (0.5% DMSO, Control), BPTES, C-968, CB-839, or CPI-613 at 37°C for 2 h. The inhibitor concentrations were all at 50 μM.

Table S1

Table S1. Screening of potent and selective mitoATP inhibitors by MitoATP-nFCM.

Compound	Molecular Target	Specific Inhibition?	Inhibition (%)[a]
**ATP Synthase Inhibitors**			
OligA	F_0 subunit c-ring	Yes	15-28%
BDQ	c and a subunits	Yes	46-56%
**ETC Inhibitors**			
Rotenone	Complex I	No	33-44%
Metformin	Complex I	Yes	22-35%
VLX600	Complex IV	Yes	42-51%
**TCA Cycle Inhibitors**			
BPTES	Pan-GLS	No	24-38%
C-968	Pan-GLS	No	25-36%
CB-839	GLS1-selective	Yes	19-44%
CPI-613	α-KGDH and PDH	Yes	39-58%

[a]Efficacy in reducing mitoATP levels within mitochondria isolated from breast and colon cancer cell lines.
